# Biogeochemistry of an Iron‐ and Manganese‐Rich Stratified Lake: Tasik Biru, Malaysia, as a Modern Model Habitat for the Ancient Ocean

**DOI:** 10.1111/gbi.70036

**Published:** 2025-10-11

**Authors:** Muammar Mansor, Andreas Kappler, Tomás Israel Grijalva Rodríguez, Samuel Lihan, Sergei Katsev

**Affiliations:** ^1^ Geomicrobiology, Geosciences Department University of Tuebingen Tuebingen Germany; ^2^ Cluster of Excellence: EXC 2124: Controlling Microbes to Fight Infection Tuebingen Germany; ^3^ Universidad Autónoma de Baja California Sur La Paz Baja California Sur México; ^4^ Institute of Biodiversity and Environmental Conservation Universiti Malaysia Sarawak (UNIMAS) Kota Samarahan Sarawak Malaysia; ^5^ Large Lakes Observatory University of Minnesota Duluth Duluth Minnesota USA

## Abstract

Tasik Biru is a ~70 m‐deep tropical lake in Malaysia, originating from a water‐filled open pit mine. We investigated the biogeochemistry and microbial community of the lake as a modern model habitat to the stratified ancient ocean. We found that a sharp redoxcline exists at around 50 m depth, related to the decrease of O_2_ and pH (7.2–6.8) going down into the monimolimnion. Despite being relatively sulfate‐rich (~320 μM), only a slight decrease of sulfate (to ~240 μM) was observed coupled with an increase of dissolved sulfide to 4 μM, attributed to microbial sulfate reduction in the monimolimnion. Comparatively, dissolved Fe and total Mn rose to ~50 μM in the anoxic layer with an unusual 1:1 concentration ratio. Other nutrients (PO_4_^3−^, Si) and trace metal(loid)s (As, Mo, Sb, Co, U, and V) depth profiles increased or decreased across the chemocline, indicating controls via cycling of redox‐sensitive elements. Microbial community analysis based on 16S rRNA amplicon sequencing reflects various metabolisms, from aerobic metabolisms in the mixolimnion to putative nitrite‐dependent methane oxidation (e.g., by *Methylomirabilis*) at the chemocline, to sulfate reduction, methanogenesis, and fermentation in the monimolimnion. Tasik Biru is not in steady‐state, and its anoxic water is predicted to shift from being Fe/Mn‐rich to sulfide‐rich, perhaps lending it as a model habitat to investigate biogeochemical changes from the metal‐rich Archean to the Proterozoic oceans with expanding zones of sulfide‐rich margins. An overview of the current biogeochemical cycles in the lake is presented, and open questions regarding partial sulfate consumption, methane, and Mn cycling and mineralogical distribution are highlighted to guide future studies.

## 1 Introduction

Life and Earth have coevolved over 4.5 billion years (Ga), resulting in different periods of Earth's history marked by progressive oxygenation of the atmosphere and the ocean. In the Archean eon (2.4–4 Ga), Earth was devoid of free oxygen (O_2_), and the ancient ocean was thought to be anoxic and rich in dissolved iron (Fe) and manganese (Mn). The rise of O_2_ and mild oxygenation of the surface water led to a stratified ocean with an anoxic, Fe‐rich (ferruginous) bottom water. Further biogeochemical evolution over time eventually led to a sulfide‐rich Proterozoic ocean after 2.4 Ga, especially in localized shallow or coastal regions, then finally to the O_2_‐rich ocean that we observe today in the Phanerozoic (0.5 Ga to modern) (Lyons et al. 2024).

One of the best approaches to investigate ancient environments is to study modern settings that exhibit similar biogeochemical features. For instance, several modern stratified lakes with ferruginous bottom waters were proposed as model habitats for the Archean oceans (Koeksoy et al. 2016; Swanner et al. 2020). A majority of them are temperate lakes, but tropical lakes near the equator (e.g., in Indonesia, Cameroon, Congo, and Brazil) arguably offer a number of advantages. Given the lack of strong seasonal changes, low wind speeds, and consistent temperatures year‐round, the water column stratification in tropical lakes is generally more stable, creating more stable biogeochemical conditions (Katsev et al. 2017; Swanner et al. 2020; Aprile and Darwich 2023; Janssen et al. 2024). Understanding the biogeochemical cycling across a broad range of chemical and physical conditions found in these lakes can help us understand the broad range of the biogeochemical processes that may have existed in the ancient oceans across time and space.

**FIGURE 1:**
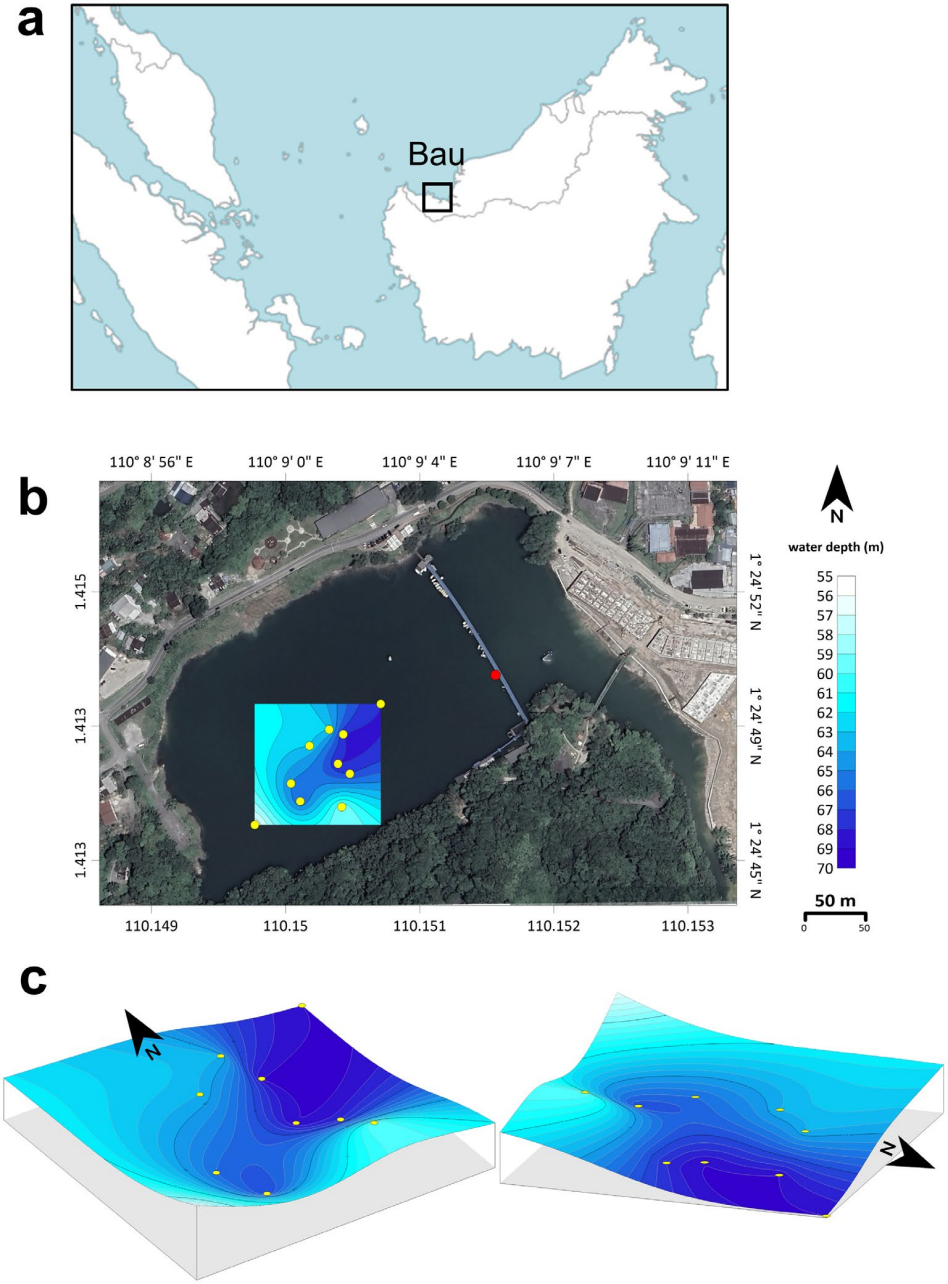
(a) Map of Malaysia and surrounding countries, with location of Bau town marked with a square. (b) Aerial view of Tasik overlain with bathymetry map and sampling locations in circles (red on the bridge and yellow at the water surface via a boat). Contour lines were created using the Kriging interpolation method based on the depths obtained from a Portable Depth Sounder. (c) Three‐dimensional models of the bathymetric map (not to scale).

Tasik Biru (“Blue Lake”) is a ~70 m deep tropical lake located in the Bau district of Sarawak, Malaysia (1.413539°N, 110.150222°E; Figure 1). Pioneering work by Sari et al. (2006) showed that the lake was stratified over three sampling campaigns in 2003 and 2004, with layer‐dependent behaviors in terms of temperature, pH, dissolved Fe, and manganese (Mn). The man‐made lake originated as an open pit that was historically mined for gold (Au), antimony (Sb), and mercury (Hg) (Schuh 1993). While the region is mostly known for its limestone‐rich karst landscape (Bau Limestone Member), intrusions in the form of granodiorite dikes (mid‐Miocene, 10–12 Mya) rich in disseminated pyrite, pyrrhotite, arsenopyrite (FeAsS), stibnite (Sb_2_S_3_), and Pb‐Sb sulfosalts are common and are the source of the economic deposits (Schuh 1993; Hutchison 2005). The Tai Parit mine (which later became Tasik Biru) is an example of an epithermal Au deposit hosted between the Pedawan shales (Late Jurassic to Turonian), the Bau Limestone member (Late Jurassic to Cenomanian), and the Krian gritstone (Late Jurassic) with a high arsenic (As) and Mn anomaly (up to 100,000 ppm) (Schuh 1993). Mining continued until the site was flooded in 1921. The lake was drained and mined again from 1990 to 1997. Following its closure, the mine naturally filled up with water thought to originate from rain and groundwater springs that percolate from the karst (Gold Mine Turned Tourist Attraction 2019; https://www.sarawaktribune.com/gold‐mine‐turned‐tourist‐attraction/). Today, the lake is known as a recreation spot with a distinct bluish color thought to come from residual As that reaches the surface water.

We conducted a biogeochemical investigation of Tasik Biru to identify its suitability as a modern model habitat to the ancient ocean. We find that the lake has an unusual Fe/Mn‐rich monimolimnion with a high sulfate concentration, suggesting it is not in steady‐state. With a predicted transition to a sulfide‐rich water column, mimicking the Earth's oceanic history in the Archean–Proterozoic time period, this lake may be a promising field site for the investigation of biogeochemical proxies preserved in dynamic Precambrian oceans. In addition, our research provides insight into the fate of toxic compounds (e.g., As and Sb) in this lake, which is pertinent given that development of Tasik Biru as a recreational site is being planned for the coming decade.

## 2 Materials and Methods

### 2.1 Sample Collection

A 3‐day field campaign was conducted on 10–12 June 2024. Depth was determined via a handheld sonar depth finder. Temperature and conductivity were measured in situ by a YSI Castaway CTD. Water was obtained from depth via a peristaltic pump and passed through Tygon tubing for 5–10 min. The water was filled into a container and allowed to overflow before pH and dissolved O_2_ measurements using a WTW 3630 multimeter hand probe. The water was then filtered inline through a 0.22 μm polyethersulfone Luer‐Lok filter into three aliquots: (a) “acidified”: 15 mL acidified with 20 μL of 6 M HCl, (b) “Zn‐fixed”: 15 mL fixed with 0.5 mL of 1 M ZnCl_2_, and (c) “not fixed”: 15 mL of sample. In addition, 2–4 L of unfiltered water from each depth was collected into plastic containers that were prerinsed with 1–2 volumes of water for DNA analysis. The water was then filtered on site through a 47 mm diameter 0.22 μm pore size multicellulose ether filters (product # GSWF047S6) fitted onto 500 mL filter towers that were cleaned with isopropanol pads in between samples. After filtration of 1.5–2 L of water, the filters were transferred into sterile 50 mL tubes.

Sediment sample from 58 m depth (under anoxic water) was obtained using a 2 kg Eckman grab sampler and aliquoted into 50 mL tubes at the surface. A soil grab sample approximately two meters from the southwest shore was obtained for comparison.

All samples were stored in an ice‐filled cold box on site. Once back at the accommodation, Zn‐fixed water samples, filter papers, sediment, and soil were kept frozen at −20°C. Filter papers, sediment, and soil were kept on ice during transport to Universiti Malaysia Sarawak (UNIMAS) for DNA extraction (see below). All other samples (including aliquots of sediment and soil) were also kept cold during transport to the University of Tuebingen until the rest of the analysis.

### 2.2 Dissolved Phase Analysis

Acidified samples—dissolved Fe^2+^ and total Fe were measured via the modified ferrozine method (Hegler et al. 2008), employing 40 μL of samples instead of 20 μL to improve the detection limit. Dissolved organic carbon (DOC) and total nitrogen were measured with a TOC/N analyzer (multi N/C 2100S, Analytik Jena AG, Germany). Dissolved metals were measured on the ICP‐MS following dilutions with 2% HNO_3_. Dissolved silica was measured using the molybdenum blue method (Schad et al. 2022).

Zn‐fixed samples—dissolved sulfide was measured via the methylene blue method (Bronner et al. 2023). Briefly, 250 μL of sample was mixed with 100 μL of ADMA (asymmetric dimethylarginine), 250 μL of H_2_O, and 100 μL of 0.38 M FeCl_3_ solution. The mixture was incubated for 30 min before the absorbance was measured at 665 nm.

Not fixed samples—dissolved anions (sulfate, chloride, and nitrate) were measured on the ion chromatogram (Metrohm 930 Compact IC Flex). Blank prepared the same way as the samples yielded values below the instrument detection limit. Dissolved ammonium (NH_4_^+^), nitrate (NO_3_^−^), and nitrite (NO_2_^−^) were measured using a flow injection analysis system (3‐QuAAtro; Bran+Lübbe, Norderstedt, Germany). Dissolved phosphate was measured using the phosphomolybdate colorimetric assay (Bronner et al. 2023).

Analytical errors for all analyses were always less than 10% (typically less than 5%) based on standards measured in the same analytical session. Detection limits for all analyses are provided in Table S1.

### 2.3 Density and Stability Calculations

**FIGURE 2:**
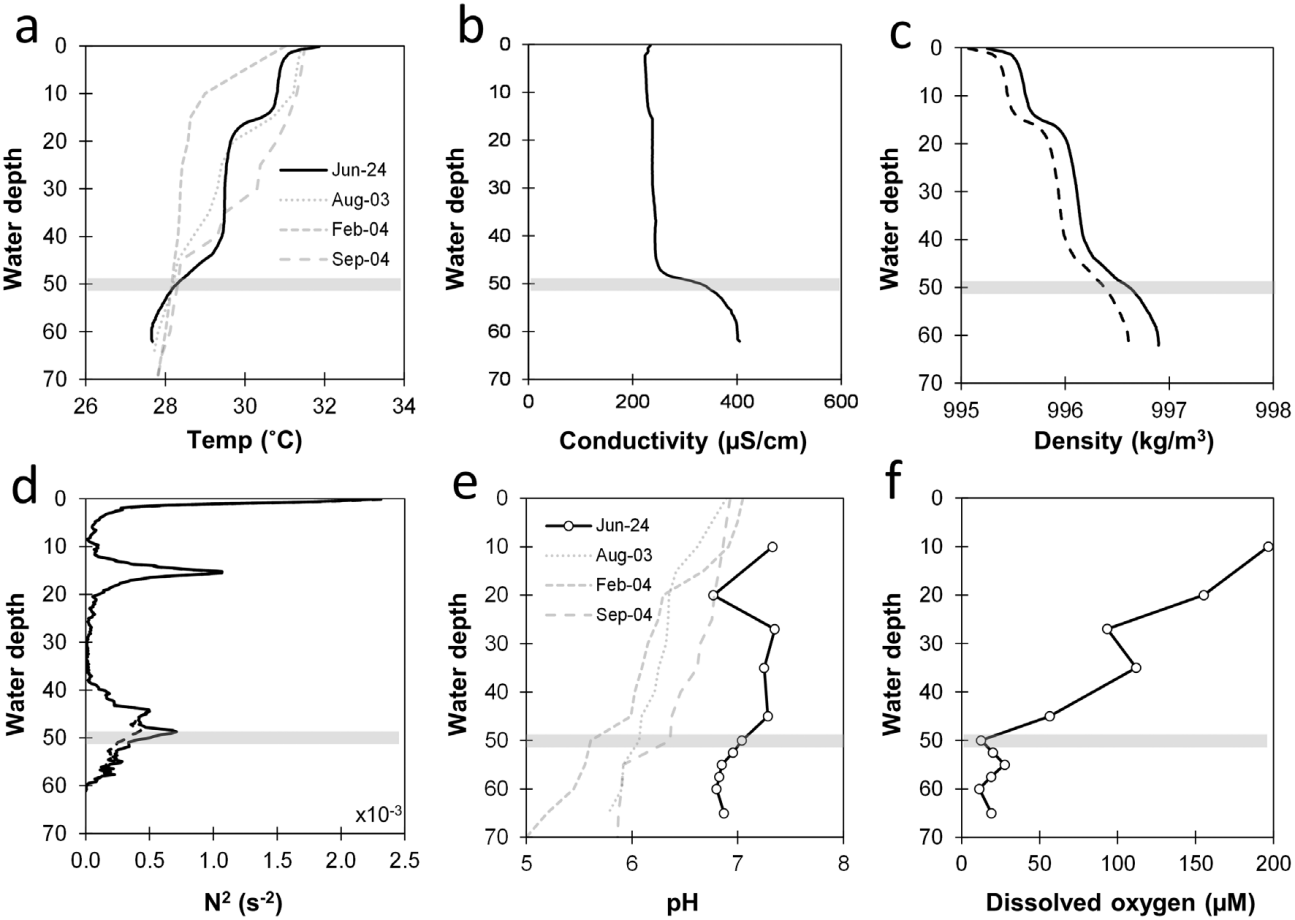
Depth profiles of (a) temperature‐compensated specific conductance, (b) conductivity, (c) calculated water density, (d) calculated Brunt‐Väisälä square of the stability frequency, (e) pH and (f) dissolved O_2_ (DO). Gray horizontal bars mark the inferred chemocline. In (c) and (d), dashed lines denote the calculated profiles for zero salinity to illustrate salinity effects on the gradients. In (e) and (f), dashed gray lines denote temperature and pH data collected by Sari et al. (2006) for comparison. Dissolved O_2_ was converted from % saturation to micromolar assuming 236 μM of O_2_ at saturation at 30°C. Dissolved O_2_ below 50 m may be overestimated due to the method limitation.

Water density ρ was calculated from the measured temperature and conductivity profiles using the *sw_dens* function of the Matlab SeaWater 3.0 library. Conductivity (mS/cm) was converted to salinity (PSU) using a conversion factor of 0.9 PSU/(mS/cm), which was estimated based on the measured concentrations of ionic and nonionic species and the requirement of charge balance. The contributions of salinity to the water density and its gradient were minor (Figure 2). The square of the Brunt‐Väisälä stability frequency $N^{2}=-\frac{g}{\rho }\frac{\mathrm{d\rho}}{\mathrm{dz}}$, which characterizes the strength of the density gradient, was computed from the density profile using the *sw_bfrq* function of the same library.

### 2.4 Geochemical Modeling

Modeling of the saturation state of minerals across water column depth was performed in Phreeqc version 3.6.2 using the Minteq version 4 database, amended with log *K*_*sp*_ of kutnahorite [CaMn(CO_3_)_2_; log *K*_*sp*_ = −20.77] (Mucci 2004). The concentrations of inorganic carbon (e.g., HCO_3_^−^) were calculated at each depth, assuming charge balance.

### 2.5 Solid Phase Analysis

The samples were freeze dried at the University of Tuebingen. The soil sample was pre‐sieved to < 450 μm to remove pebbles and root debris. Subsequently, both soil and sediment samples were powdered using a mortar and pestle and sieved to < 63 μm. Powder X‐ray diffraction (XRD) was employed to identify the dominant solid‐phase mineralogy. The total carbon (C), nitrogen (N), and sulfur (S) contents were measured on the Elementar Vario CNS Analyzer.

Solid phase distribution of metals was assessed via acid extractions. The extractions were done in parallel with three different methods: (a) 1 M HCl for 2 h with intermittent shaking for sorbed species and poorly crystalline minerals (e.g., ferrihydrite, mackinawite, and siderite), (b) 6 M HCl for 2 h with intermittent shaking for crystalline minerals (including goethite, hematite, and magnetite) (Voelz et al. 2019; Lueder et al. 2020), and (c) a 3:1 mixture of concentrated HNO_3_ and HCl via microwave digestion (MARS 6) at 180°C for 15 min to obtain total acid‐extractable elements (the fractions described above plus pyrite and some clays). All extractions employed ~100 mg of powdered material and 10 mL of extractant. After extractions, the samples were centrifuged at 12,000 *g* for 5 min. The supernatants were then transferred into 15 mL tubes for storage at 4°C. The microwave extracts were diluted in water 4‐fold for long‐term storage. The concentrations of extractable Fe and other metals were measured via ferrozine and ICP‐MS, respectively, as described above. Three fractions were then defined for discussion: (a) poorly crystalline (1 M HCl), (b) crystalline (6 M HCl—1 M HCl), and (c) recalcitrant (total acid‐extractable—6 M HCl). Blanks were processed in parallel and were below detection limits except for Na.

### 2.6 Microbial Community Analysis

DNA from water samples collected on filter papers and wet sediment at 58 m depth was extracted at UNIMAS for microbial community analysis based on 16S rRNA. Filter papers were aseptically cut using forceps and transferred into extraction tubes. DNA was extracted using the Macherey‐Nagel NucleoSpin Soil kit using Buffer SL2, and the yield was determined fluorometrically (Quantus Fluorometer). No DNA was able to be extracted from blank filters. Extracted DNA was shipped to Patriot Biotech Sdn. Bhd. for 16S rRNA amplicon sequencing. Subsequent sample preparation and data analysis are as described below by the company.

The 16S rRNA V3 (bacteria only) and V4‐V5 (bacteria and archaea) hypervariable regions were amplified using the primer pair 341F CCTACGGGNGGCWGCAG and 534R ATTACCGCGGCTGCTGG and the primer pair 515F GTGYCAGCMGCCGCGGTAA and 926R CCGYCAATTYMTTTRAGTTT, respectively (Klindworth et al. 2013; García‐López et al. 2020). An additional five bases of inline barcode and partial Illumina adapter were incorporated at the 5′ end of the primers to enable inline barcoding (Glenn et al. 2019). Different samples were amplified using different combinations of the forward and reverse inline primers. PCR was performed using REDiant II PCR Master Mix (Apical Scientific, Malaysia) using the PCR profile of: 95°C for 2 min followed by 30 cycles of 95°C for 15 s, 50°C for 30 s, and 72°C for 30 s.

The barcoded amplicons were subsequently visualized on gel and purified using 0.8 × vol. of SPRI bead. The purified amplicons were used as the template for 8 cycles of index PCR to incorporate the complete Illumina adapter and Illumina‐compatible dual‐index barcodes. The constructed libraries were subsequently size‐selected using 0.8 × vol of SPRI bead and pooled into a single tube. Quantification of the pooled libraries used Denovix high sensitivity assay. Sequencing of the pooled libraries was performed on a NovaSEQ6000 (Illumina, San Diego) using the 2 × 150 bp paired‐end sequencing configuration.

For data analysis, demultiplexing and primer trimming of the raw paired‐end reads, we used cutadapt v1.18 (Martin 2011). The trimmed reads were subsequently merged using fastp v0.21 (Chen et al. 2018). The processed reads were imported into QIIME2 v.2023.9 (Bolyen et al. 2019) and denoised into Amplicon Sequence Variant (ASV) with dada2 v.1.26.0 (Callahan et al. 2016). Taxonomic assignment of the ASV used q2‐feature‐classifier (Bokulich et al. 2018) that has been trained on the latest GreenGenes2 (McDonald et al. 2024).

ASVs (amplicon sequence variants) with taxonomic assignment to at least the phylum level were selected for subsequent analysis. Following data rarefaction to the sample with the lowest number of reads, alpha diversity analysis was conducted in QIIME2. Diversity indices including Chao1, ACE, Shannon, Simpson, and Faith_PD were calculated to assess within‐sample diversity. For beta diversity analysis, distances based on Jaccard, Bray Curtis, weighted UniFrac, and unweighted UniFrac were computed using the rarefied dataset. Additionally, the unrarefied dataset was utilized for the calculation of Robust Aitchison PCA (RPCA) and compositionally aware phylogenetic beta‐diversity (phylo‐RPCA). Both ASV table and taxonomic classification table were exported using QIIME2 tools into tab‐separated values (tsv format).

The tables were then formatted with Python to generate inputs compatible for visualizing microbial community via Sankey diagrams (in the web version of Pavian https://shiny.hiplot.cn/pavian/; Breitwieser and Salzberg 2020) and heatmaps. Functional prediction of each ASV was performed using FAPROTAX software (http://www.loucalab.com/; Louca et al. 2016).

## 3 Results

### 3.1 Depth Profiles of Temperature, Conductivity, and pH

Persistently stratified lakes are typically divided into the mixolimnion (O_2_‐rich upper layer), the monimolimnion (O_2_‐poor bottom water), and the metalimnion that separates the two layers, which is typically the region with the steepest density and chemical concentration gradients. To guide our sampling depths, we first determined the temperature and conductivity profiles at 0.3 m resolution. Six temperature and conductivity profiles were collected over the sampling campaign, and they all overlapped with only small differences. Hence, we only show one representative profile of each in Figure 2. Temperature decreased from 32°C to 28°C to the bottom and was in general agreement with those measured by Sari et al. (2006), albeit with differences in the depth gradients (Figure 2a). In our campaign, three layers could be identified, separated by steep temperature gradients: 0–15 m, 15–45 m, and below 45 m. In contrast, the conductivity profile indicated only two separate layers, separated by a steep gradient at around 50 m (Figure 2b). Conductivity increased from 265 μS/cm at the top to 427 μS/cm at the bottom. Dissolved chloride was below the detection limit of the ion chromatogram (< 0.3 mM after accounting for the dilution factor).

The pH values and dissolved O_2_ were measured at coarser depth intervals of 2.5–10 m. The pH values were relatively constant at 7.2–7.3 above 50 m (with one outlier at 20 m) before decreasing to 6.8–7.0 below 50 m. This compares with the data of Sari et al. (2006), which showed a much steeper gradient down to a pH value of 5 at the deepest depth (Figure 2c). Meanwhile, dissolved O_2_ was ~200 μM at 10 m, before showing a steady decrease with depth and then remaining stable at 10–20 μM below 50 m (Figure 2d). It must be noted that pH and dissolved O_2_ were measured using hand probes after pumping water to the surface. Gas bubbles could be observed in the tubing during pumping to the surface, which may be caused by the degassing of gases such as CO_2_ that could raise the pH:

(1) $${{\mathrm{HCO}}_{3}}^{-}+H^{+}➔{\mathrm{CO}}_{2}\left(g\right)+H_{2}O$$

Furthermore, atmospheric O_2_ could diffuse into the water during measurements, which would have raised the dissolved O_2_. Hence, the pH and dissolved O_2_ data should be treated as approximate, pending further confirmation by in situ measurements at depth. Nonetheless, the data indicated the existence of a separate mixolimnion and monimolimnion separated by a chemocline layer at around 50 m.

### 3.2 Depth Profiles of Nutrients: Carbon (C), Nitrogen (N), Phosphorus (P), and Silica (Si)

**FIGURE 3:**
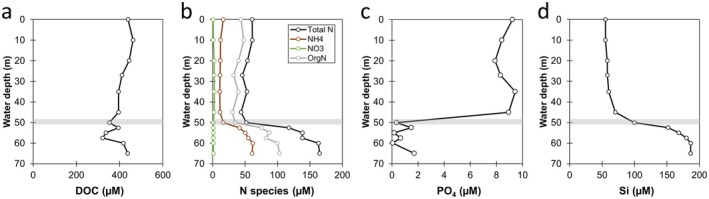
Depth profiles of dissolved (a) organic carbon (DOC), (b) nitrogen species, (c) phosphate and (d) silica. Gray horizontal bars mark the inferred chemocline. Organic N was calculated by subtracting NH_4_^+^ and NO_3_^−^ from total N.

The concentrations of dissolved organic carbon (DOC) showed no clear depth‐dependent trend, ranging from 320 to 460 μM (Figure 3a). This is on the lower range of 400–1050 μM DOC determined globally for lakes (Massicotte et al. 2017). Meanwhile, total N remained stable at 45–60 μM before showing a clear increase to 165 μM below 50 m. Much of the increase was attributable to the increase in NH_4_^+^, with the remainder attributable to organic N. Nitrate was barely detectable above the detection limit (1–2 μM), while NO_2_^−^ was not detectable (detection limit ≈1 μM) (Figure 3b). Dissolved PO_4_^3−^ showed a clear trend of decreasing with depth, with values of 8–10 μM above 50 m and < 2 μM below it (Figure 3c). Meanwhile, dissolved Si showed a slow increase from 55 to 70 μM with depth above the chemocline, before a rapid increase below 50 m up to 190 μM (Figure 3d).

### 3.3 Depth Profiles of Major Redox‐Sensitive Elements: Sulfate, Sulfide, Iron, and Manganese

**FIGURE 4:**
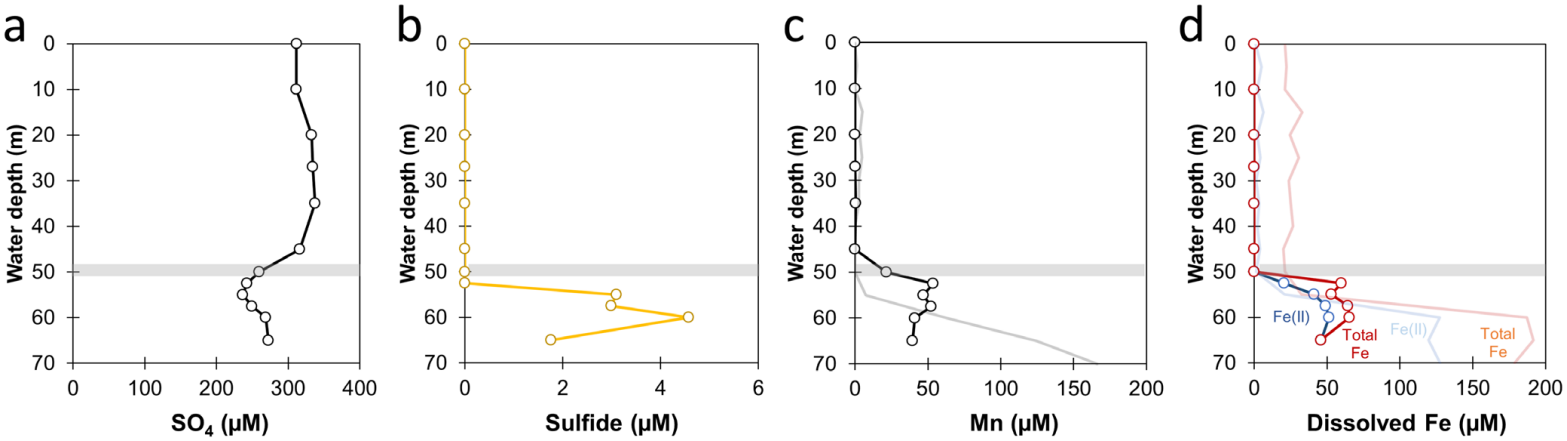
Depth profiles of dissolved (a) sulfate, (b) sulfide, (c) manganese (Mn), and (d) Fe species. Gray horizontal bars mark the inferred chemocline. Dissolved Mn and Fe profiles (lighter shades) collected by Sari et al. (2006) are included for comparison.

As dissolved O_2_ decreased significantly below 50 m, other electron acceptors such as sulfate, Fe(III), and Mn(IV) would be used by microorganisms for anaerobic respiration. Sulfate was the most abundant electron acceptor in the dissolved phase, with concentrations ~320 μM in the mixolimnion and a slight depletion to 240–270 μM below the chemocline (Figure 4a). This suggests the activity of microbial sulfate reduction to sulfide at and below the chemocline. Indeed, sulfide increased up to 4.6 μM from 52.5 to 60 m, followed by a decrease to 1.8 μM at 65 m (Figure 4b). The presence of sulfide was consistent with the rotten egg smell emanating from the water at those depths during sampling.

In comparison, dissolved Fe and Mn were below detection limits in the mixolimnion before increasing rapidly below the chemocline (Figure 4c,d). Dissolved Mn increased first at 50 m, followed by Fe at 52.5 m and finally sulfide at 55 m. Dissolved Mn increased to 41–54 μM while dissolved total Fe increased to 53–65 μM with depth. These values were lower than the maximum values measured previously at 166 μM (Mn) and 130 μM (total Fe) by Sari et al. (2006). The percentages of dissolved Fe^2+^ increased from 34% just below the chemocline to 100% at depth. The presence of dissolved Fe^2+^ was supported by the observation that the water turned orange within 30 min of air exposure, indicative of Fe^2+^ oxidation and subsequent precipitation as Fe(III) (oxyhydr)oxides (Figure S1).

### 3.4 Depth Profiles of Other Elements

**FIGURE 5:**
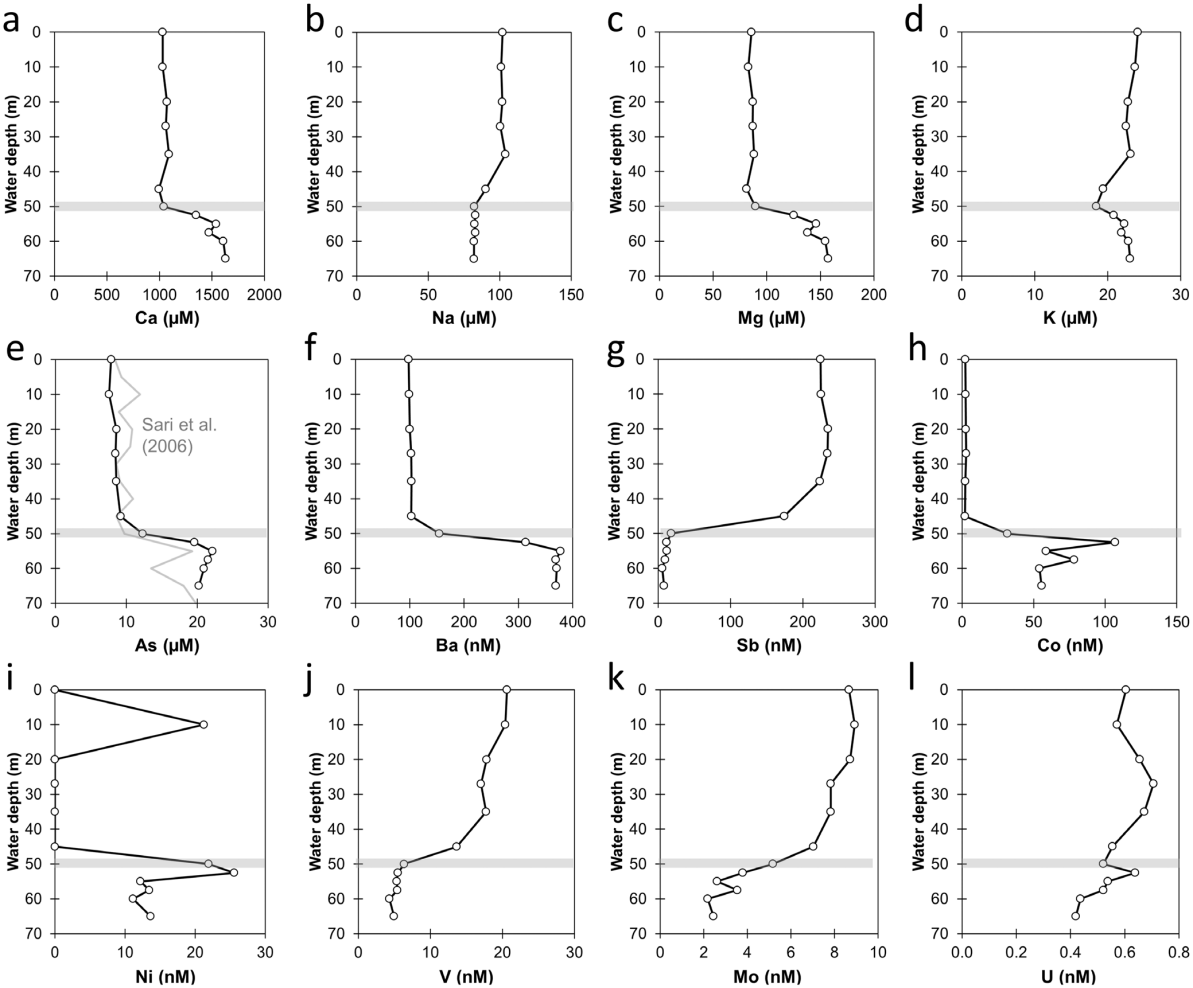
Water column depth profiles of Ca, Na, Mg, K, As, Ba, Sb Co, Ni, V, Mo, and U.

Within the water column, the highest concentrations were observed for Ca (995–1630 μM), Mg (81–157 μM), Na (82–104 μM), K (18–24 μM), and As (8–22 μM) (Figure 5). The depth profile of As in this field campaign was nearly identical to the one measured by Sari et al. (2006). Other elements had lower concentrations in the nanomolar range, including Ba (98–378 nM), Sb (6–235 nM), Co (2–107 nM), Ni (11–26 nM), V (4–20 nM), Mo (2–9 nM), and U (0.4–0.7 nM). Zinc (< 3 nM), Cu (< 18 nM), and Se (< 60 nM) were below the ICP‐MS detection limits.

The depth profiles of the elements could be divided into three categories: (1) no clear trend with depth—K, (2) increasing below the chemocline—Ca, Mg, As, Ba, Co, Ni, and (3) decreasing below the chemocline—Na, Sb, V, Mo, and U. For Co and Ni, these two elements showed their highest concentrations at or just below the chemocline, before decreasing deeper into the monimolimnion. These trends are influenced by multiple complex factors including pH, redox changes mediated by microorganisms, mineral formation and dissolution (e.g., calcite, clays, barite, and Fe minerals), and adsorption/release of the dominant redox species onto/from mineral surfaces, as will be discussed later.

### 3.5 Solid Phase Geochemistry

**FIGURE 6:**
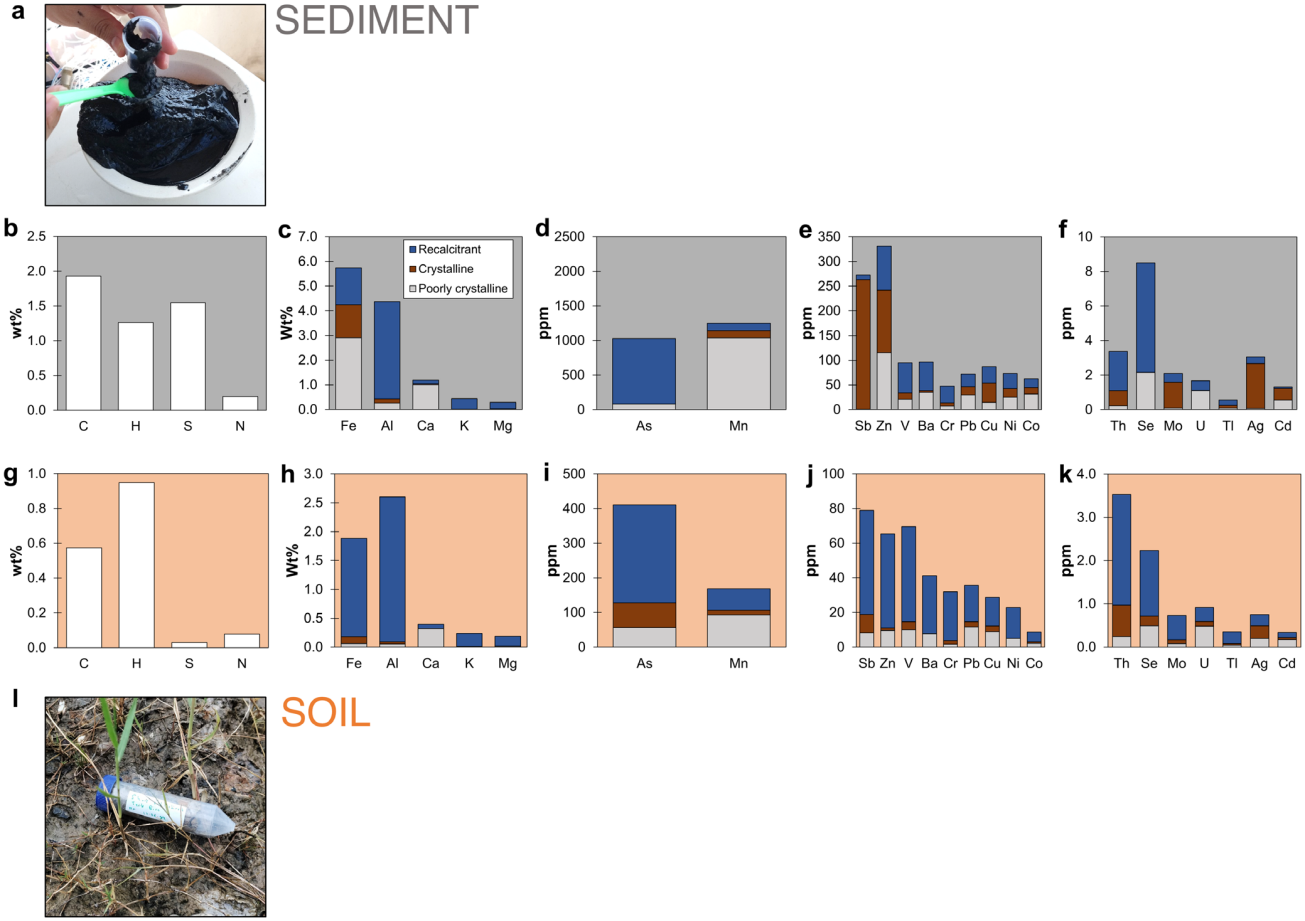
Photos (a and l) and solid phase contents of various elements in (a–f) lake sediment collected at 58 m depth and (g–k) soil for comparison. Note the higher *y*‐axis values for the lake sediment compared to the soil and also the different *y*‐axis values between all the plots.

Solid phase contents of lake sediment collected at 58 m depth were characterized and compared to a soil sample collected from the shore of Tasik Biru (Figure 6). All data are reported in Table S2. The soil sample was mud‐like and contained orange spots with thin gray layers. In comparison, the lake sediment had a slush‐like consistency with black colorations, which turned slightly gray upon exposure to O_2_. Analysis via XRD indicated the presence of quartz and a clay‐like phase similar to kaolinite in both the soil and lake sediment samples (Figure S2).

The lake sediment sample contained high C (1.9 wt%) and S (1.5 wt%) (Figure 6b). The most abundant element in the sediment was Fe (5.7 wt%) followed closely by Al (4.4 wt%) and then Ca, K, and Mg (0.3–1.2 wt%) (Figure 6c). Arsenic content (1030 ppm) was similar to Mn (1250 ppm) (Figure 6d). All other elements were lower in the lake sediment with a concentration range of 62–273 ppm (Sb, Zn, V, Ba, Cr, Pb, Cu, Ni, and Co) (Figure 6e) or 0.6–8.5 ppm (Th, Se, Mo, U, Tl, Ag, and Cd) (Figure 6f). Solid phase Na is not reported due to high blanks during the extraction.

The soil sample contained lower C (0.6 wt%) and much lower S (0.03 wt%) compared to the lake sediment (Figure 6g). Aluminum (2.6 wt%) was the most abundant element, followed by Fe (1.9 wt%) and then Ca, K, and Mg (0.2–0.4 wt%) (Figure 6h). Intriguingly, As content (410 ppm) exceeded Mn (170 ppm) by 2.4× (Figure 6i). All other elements were found at lower concentrations of 9–79 ppm (Sb, Zn, V, Ba, Cr, Pb, Cu, Ni, and Co) (Figure 6j) or 0.3–3.5 ppm (Th, Se, Mo, U, Tl, Ag, and Cd) (Figure 6k).

In comparison to soil, all elements except Th were enriched in the lake sediment by a factor of 1.3–51.5×. The most extreme enrichment was observed for S (51.5×), Mn (7.4×), and Co (7.1×). The lowest enrichments were observed for Al, Mg, V, Cr, and Tl (≤ 1.7×).

Extractions with acids of different strengths indicated the partitioning of these elements within various solid phase fractions, which can be used to infer their mineral association and corresponding likelihood to be released to the dissolved phase. Compared to soil, differences in solid phase partitioning were observed for Fe, As, Sb, Zn, Co, Mo, and Ag in the lake sediment.

In soil, most of the Fe resided in the recalcitrant fraction (90%). The remaining HCl‐extractable fraction consisted solely of oxidized Fe(III). In contrast, a large percentage of Fe in the lake sediment resided in the poorly crystalline (51%) and crystalline fractions (24%) (Figure 6c,h). All of the Fe in the poorly crystalline fraction was in the reduced Fe(II) form, while nearly all the Fe in the crystalline fraction was in the form of oxidized Fe(III). The samples were not frozen immediately, and handling in the atmosphere could lead to oxidation and underestimation of the Fe(II) content. Nevertheless, the detection of solid‐phase Fe(II) and Fe(III) indicates active Fe redox cycling in the sediment.

The same partitioning trend could be observed for Sb, Zn, Co, Mo, and Ag. These elements mostly resided in the recalcitrant fractions in the soil (35%–83%), while the poorly crystalline or crystalline fractions were more abundant in the lake sediment (72%–97%). This is suggestive of repartitioning mediated by active redox cycling or association with sulfide. Furthermore, the poorly crystalline fraction of Sb, Mo, and Ag was very low in the lake sediment (0.2%–5%), which likely indicated that the elements had been mobilized to the water column.

Intriguingly, As showed the opposite behavior in which the recalcitrant fraction increased in relative abundance in lake sediment (92%) compared to the soil (69%). This indicated that either As that resided in the poorly crystalline or crystalline fraction had been released to the water column at depth or that As had been repartitioned into more recalcitrant phases such as clays or pyrite. Partitioning differences for the other elements were relatively small compared to those that have been mentioned above and will not be focused on here.

### 3.6 Microbial Community Analysis

**FIGURE 7:**
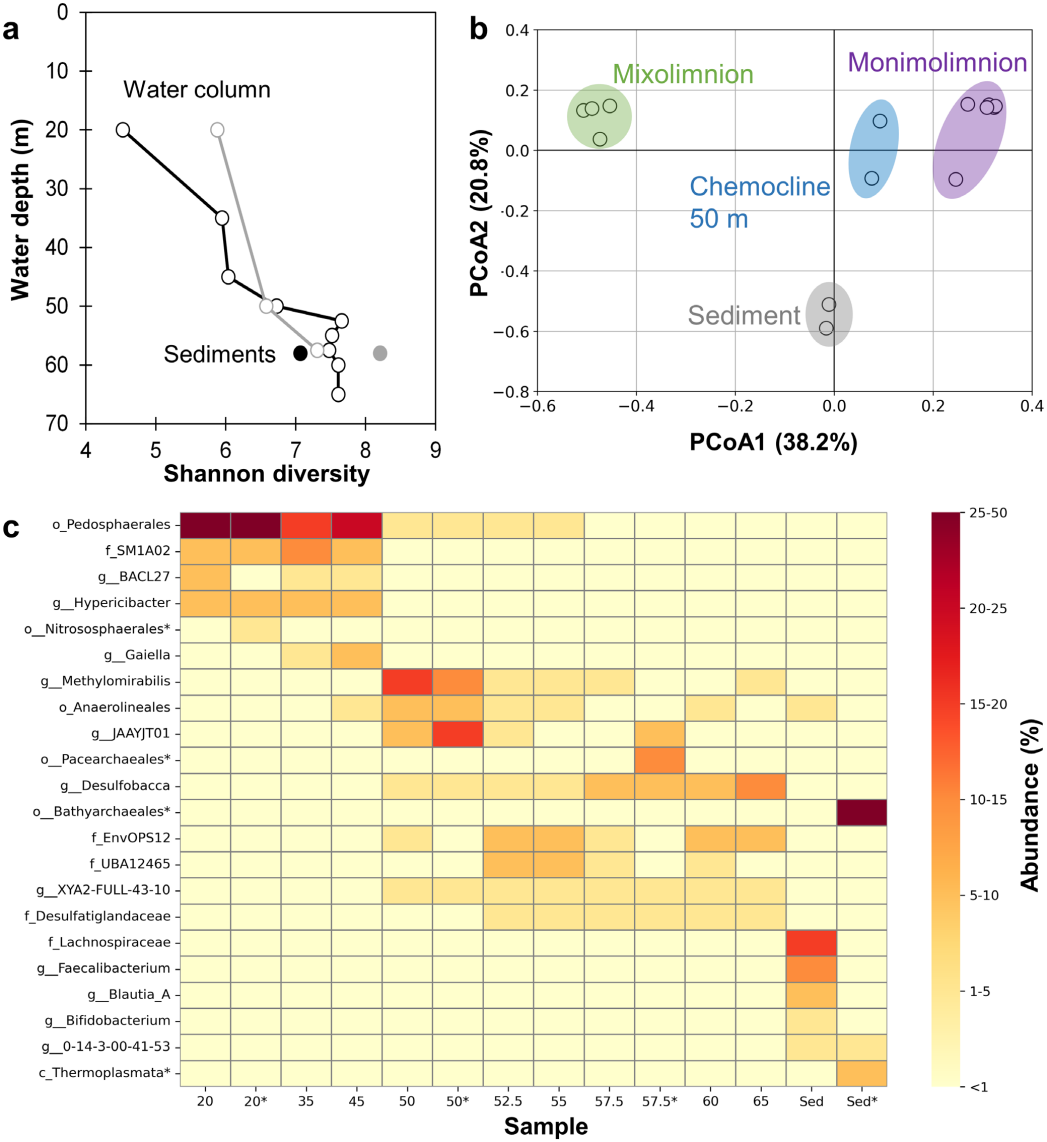
(a) Shannon diversity index across depth in the water column and sediment. Black lines and markers are dataset from bacterial‐specific primers. Gray lines and markers are dataset from universal primers. (b) Principal coordinate analysis (PCoA) biplot based on the Bray–Curtis dissimilarity metric. (c) Heatmap of top ASVs showing their distribution across depth. Samples marked with an asterisk (*) are sequenced using universal primers, where archaeal ASVs (also marked with an asterisk on the *y*‐axis) can be observed in addition to bacterial ASVs.

16S sequencing with both bacterial‐specific and universal primers showed that the Shannon diversity increased with depth before remaining nearly constant below 50 m (Figure 7a; Figure S3). Principal coordinate analysis grouped the microbial community into four different groups: (1) mixolimnion (20, 35, and 45 m), (2) chemocline (50 m), (3) monimolimnion (52.5, 55, 57.5, 60, and 65 m), and (4) lake sediment community (Figure 7b). This grouping mirrored the assignment of the water column and sediment based on physical and geochemical data and was also reflected in the relative abundances heatmap of the microbial community across depth (Figure 7c).

From here on, we focus on the top five Amplicon Sequence Variants (ASVs) within each group based on their relative abundances down to the genus level when possible (Figure 7c, Figure S4).

In the mixolimnion, the bacterial community was dominated by the order Pedosphaerales (16%–47%), the family SM1A02 (6%–14%), and the genera BACL27 (2%–6%), *Hypericibacter* (5%–8%), and *Gaiella* (0.7%–6%). In general, these ASVs are aerobic heterotrophs, consistent with their presence in the oxygenated mixolimnion (Appendix S1).

The monimolimnion bacterial community was dominated by the genus *Desulfobacca* (3%–13%), the family EnvOPS12 (5%–8%), UBA12465 (UBA = Uncultivated Bacteria and Archaea; 1%–6%), and Desulfatiglandaceae (3%–13%), and the order XYA2‐FULL‐43‐10 (2%–5%). Both *Desulfobacca* and Desulfatiglandaceae are known sulfate reducers, while the rest are associated with poorly known metabolisms or symbiotic lifestyles (Appendix S1).

Interestingly, the chemocline bacterial community was distinct from both the mixolimnion and the monimolimnion, with high abundances of the genus *Methylomirabilis* (18%) and the family JAAYJT01 (5%). The genus *Methylomirabilis* is known for catalyzing nitrite‐dependent methane oxidation (Wu et al. 2011; Versantvoort et al. 2018; Yao et al. 2024). This implies the presence of nitrite and methane (CH_4_) in the water column, even though they were either below detection limit (NO_2_^−^ < 1 μM) or not measured (CH_4_). In comparison, the genome of JAAYJT01 has been recovered from ancient groundwater, implicating a mode of life via fermentation of oligosaccharides with corresponding H_2_ production (Ruff et al. 2023). The chemocline layer also contained mixtures of communities from the mixolimnion (e.g., Pedosphaerales) and the monimolimnion (e.g., *Desulfobacca*, members of the UBA and Anaerolinea) at lower relative abundances.

Finally, the sediment bacterial community was dominated by anaerobic fermenters of complex organic carbon such as the family Lachnospiraceae (28%) and the genera *Faecalibacterium* (11%), *Blautia_A* (9%), *Bifidobacterium* (4%), and 0‐14‐3‐00‐41‐53 (3%) (Appendix S1).

The indication of an anoxic carbon cycle, perhaps with methane as a substrate for methane‐oxidizing bacteria such as *Methylomirabilis* at the chemocline, motivated us to perform further 16S rRNA amplicon sequencing to detect archaea that are known to be methanogens and anaerobic heterotrophs (Baker et al. 2020). Four representative samples were therefore chosen for sequencing via universal 16S rRNA primers (due to the absence of archaeal‐specific primers and Malaysia's biological export restrictions): mixolimnion—20 m, chemocline—50 m, monimolimnion—57.5 m, and sediment. The results showed that archaea were present throughout the lake. They comprised a minor fraction of the community at the mixolimnion (4.3%) and the chemocline (2.1%). They were much higher at the monimolimnion (11.6%) and made up a significant portion of the microbial community in the sediment (39.6%) (Figure S4). The bacterial community determined via 16S sequencing based on the two primer sets (bacterial only versus universal) was mostly similar apart from the sediment, most likely due to primer biases (Appendix S3).

The order Nitrososphaerales—known as aerobic ammonia‐oxidizers—dominated in the mixolimnion (92% of total archaea). Meanwhile, the monimolimnion was dominated by the Pacearchaeales (86% of total Archaea), which are associated with symbiotic lifestyles and small genomes that encode for fermentation and CO_2_ fixation. The sediment was mostly comprised of a mixture of Bathyarchaeales (69%) and the class Thermoplasmata (19%), known to be complex carbon degraders. Lastly, the chemocline exhibited a more diverse archaeal community representing mixtures of the above, comprising Pacearchaeales (28%), Bathyarchaeales (23%), Nitrososphaerales (9%), the class Thermoplasmata (4%), and others with lower abundances (36% total) (Appendix S2; Figure S5). Intriguingly, there is a lack of abundance of known methanogens within the archaeal community.

### 3.7 Targeted Functional Prediction

To further identify putative microbial metabolisms, we performed functional prediction using FAPROTAX. The prediction from FAPROTAX was overall in line with expectations, with a relative dominance of aerobic chemotrophy and oxygenic photoautotrophy in the upper water column, nitrite respiration and methane/organic degradation at the chemocline, and sulfate reduction and fermentation in the deeper water column and sediments (Figure S6). We further coupled the FAPROTAX prediction with a targeted search of microbial ASVs that are known to be involved in redox cycling of key elements, as summarized below.

Light‐dependent CO_2_ fixation—oxygenic photosynthesizing cyanobacteria (family *Cyanobiaceae*; Salazar et al. 2020) was found at low abundances of < 3.1% throughout the water column and the sediment, with the highest abundance at 20 m depth. The green sulfur bacteria of the genus Chlorobaculum were present at < 0.2% in the monimolimnion. These bacteria are capable of anoxygenic photosynthesis in which reduced sulfur compounds or Fe(II) replace water as the electron donor (Chew et al. 2007).

Iron—Fe(II)‐oxidizing microorganisms (FeOM) within the family *Ferrovaceae* were present at < 1% abundances in the water column, where they might catalyze (dark) Fe(II) oxidation with O_2_ or nitrate (Grettenberger et al. 2020). Fe(III)‐reducing microorganisms (FeRM) from the order Geobacterales, containing well‐known Fe(III)‐, S^0^‐ and Mn(IV)‐reducers such as *Geobacter* species (Caccavo et al. 1994; Lovley et al. 2004; Flynn et al. 2014), were found at less than 0.3% abundances in the monimolimnion. The family *Magnetospirillaceae* could be found at < 0.1% abundances in the mixolimnion, where they could be involved in intracellular magnetite (Fe_3_O_4_) formation (Amor et al. 2020).

Sulfur—sulfate‐reducing microorganisms (SRM) from the *Desulfobacca* and Desulfatiglandaceae taxonomy dominated in the monimolimnion (3%–13%). In contrast, sulfur‐oxidizing microorganisms (SOM) were generally low in abundance. The genus *Sulfuricurvum* (0.2%–2%) and *Sulfuricella* (0.1%–2.3%) were present in the chemocline and the monimolimnion, where they are capable of (dark) anaerobic or microaerophilic oxidation of reduced sulfur compounds (Kodama and Watanabe 2004; Kojima and Fukui 2010; Han et al. 2012). Other SOM (e.g., *Sulfurimonas*, *Sulfuritalea*, and *Parasulfuritortus*) were found at < 0.2% abundances in the water column.

Manganese—diverse microbial species are known to catalyze Mn oxidation or reduction (Wang et al. 2022). Manganese(IV)‐reducing microorganisms (MnRM) within the order Geobacterales, the genus *Sulfurimonas*, and the archaea *Candidatus* Methanoperedens were detected at < 0.3% abundances throughout the lake. *Candidatus* Methanoperedens could reduce Mn(IV) coupled to methane oxidation (Leu et al. 2020). *Sulfurimonas* could couple Mn reduction to sulfide oxidation (Henkel et al. 2019). Manganese‐oxidizing microorganisms (MnOM) capable of Mn oxidation via light (e.g., *Chlorobium*; Daye et al. 2019) or aerobically (e.g., *Candidatus* Manganitrophaceae; Yu et al. 2022) were not detected.

Methane—the genus *Methylomirabilis* is the dominant methane‐oxidizing bacteria at the chemocline (18%). Other methane‐oxidizing bacteria such as *Methylomonadaceae*, *Methylocystis*, and *Methylococcaceae* (Bowman 2006; Deng et al. 2024) and methanogenic archaea such as Methanomicrobia, Methanosarcina, and Methanomassiliicoccales (Paul et al. 2012; Baker et al. 2020) were generally present at low abundances (< 0.5%) throughout the lake.

## 4 Discussion

One of the goals of this study is to investigate whether Tasik Biru could serve as a model habitat for biogeochemical processes in the ancient oceans. Below, we first discuss the stratification stability and long‐term chemical evolution of the lake. An overview of the operating biogeochemical cycles is presented, focusing on open questions ripe for future studies. As the lake is not in steady state, we propose that its geochemical evolution makes it interesting for studies that address the transition that happened during the Fe/Mn‐rich Archean to the more sulfide‐rich Proterozoic oceans, highlighting its utility as a model habitat for the Precambrian oceans in addition to other previously proposed analogue lakes.

### 4.1 Long‐Term Stratification and Chemical Evolution of Tasik Biru

Several arguments suggest that Tasik Biru is stably stratified. First, comparison of temperature, pH, Fe, and Mn profiles separated by two decades between our 2024 dataset and those of Sari et al. (2006) showed consistent stratification. Tasik Biru is generally exposed to low wind speeds (< 2.5 m/s) year‐round and is additionally wind‐protected from the southeast side (Figure 1). Effects of the size and shape of the lake on its stratification can be informed based on calculation of the lake's relative depth (Z_r_):

(2) $$Z_{r}=Z_{m}\times 88.6/\sqrt{A_{0}}$$

Given the Z_m_ = 70 m maximum depth and approximate surface area (A_0_) of 66,500 m^2^ (width 190 m, length 350 m, rectangular shape), we obtain a Z_r_ value of 20.6% for Tasik Biru. This is much higher than the value of 4% that was suggested as a typical threshold for stably stratified small temperate lakes, and also exceeds the Z_r_ values for most known ferruginous meromictic lakes worldwide (Swanner et al. 2020) The density gradient in Figure 2 corresponds to the Schmidt stability for the entire water column of Tasik Biru of about 2 kJ/m^2^. This is within the range of energies that winds can deliver over extended periods of time in large lakes at temperate latitudes, but is likely sufficient to maintain stable stratification in this small and sheltered equatorial lake. It is comparable, for example, to the stability values achieved during the windy dry seasons in the much larger Indonesian Lake Towuti, where the winds cannot regularly mix the lake (Pu et al. 2025).

While stratification appears to be stable, which should maintain persistent anoxia in the monimolimnion, a question remains whether the physical and chemical parameters of the Tasik Biru water column have approached steady state. The modern lake started after the mining pit was flooded in 1997, so additional calculations are needed to ascertain whether 27 years would be sufficient for chemical distributions to evolve to their stable state. As turbulent eddy diffusion is likely to be the dominant mechanism for the transport of water and solutes within the lake's monimolimnion, vertical profiles of solutes could be adjusting on a time scale sufficiently greater than the diffusion time scale of $\tau_{\text{diff}}={\tilde{x}}^{2}/\left(2K_{z}\right)$, where *K*_*z*_ is the coefficient of turbulent eddy diffusion and $\tilde{x}$ is the depth interval of interest. Determining the rates of turbulent mixing in stably stratified lakes is often difficult (Katsev et al. 2017), but an upper estimate for the monimolimnion can be often obtained from a broadly held phenomenological correlation of *K*_*z*_ with the Brunt‐Väisälä stability frequency: $K_{z}=3\times 10^{-10}N^{-2}$ (Katsev et al. 2010). For the deep water of Tasik Biru (Figure 2), this yields *K*_*z*_ on the order of 10^−6^–10^−5^ m/s^2^, which is realistic for a small and wind‐protected lake (Katsev et al. 2010; Pu et al. 2025). For the 30 m deep monimolimnion, the diffusion time scale $\tau_{\text{diff}}$ is then 1.4–14 years. Adjustment in the lake's hydrological regime, and also feedbacks between the distributions of solutes in the monimolimnion and the vertical fluxes of the corresponding chemical species from the mixolimnion and the underlying sediments (Katsev 2017), would further increase the time scale on which the lake's chemistry approaches a steady state regime. The chemical distributions in the lake thus may be reasonably assumed to be still in transition.

Indeed, comparison of data collected in 2003/2004 versus 2024 shows a clear decrease in the maximum concentrations of Fe and Mn in the water column coupled with an increase in the pH (Figures 2e and 4c,d). No data, unfortunately, are available for comparison for sulfate and sulfide. The levels of dissolved Fe, Mn, and sulfur species are governed by the balance between dissolution of exposed solid phases on the walls of the lake versus net burial into the sediment. Dissolution of metal‐ and sulfur‐rich deposits (e.g., pyrite, Krian gritstone with high Mn and As) could slow down as they are depleted or when they develop less reactive coatings, with pH increase also potentially encouraged by more exposure to the Bau Limestone Member that hosts the deposits over time. Concurrently, net burial of Fe and Mn into the sediment is supported by the observation of enriched Fe (enrichment factor, EF = 3) and Mn (EF = 7.4) in the lake sediment over soils. Sulfur also showed high enrichments in the sediment (EF = 51.5), but the sulfate profile indicates only partial consumption (Figure 4a), with the capacity for more sulfide production driven by abundant SRM. Currently, the SO_4_^2−^ profile with a trough at 55 m (Figure 4) would indicate not only a sink for sulfate (e.g., by sulfate reduction) at that depth but also a source of sulfate from deeper waters or sediments, such as by groundwater inflows through the porous limestone bedrock.

For Fe, we propose that the most likely phases being buried into the sediment are Fe sulfides. Phreeqc modeling predicted the formation of iron sulfides such as mackinawite (FeS) and pyrite (FeS_2_), while vivianite [Fe_3_(PO_4_)_2_٠H_2_O] remains undersaturated in the monimolimnion (Table S3). The sediment is enriched in S (EF = 51.5 relative to soil) and sulfide could be detected by smell when HCl was added to the sediment, suggesting the presence of acid volatile sulfides (AVS) such as poorly crystalline mackinawite (Rickard and Morse 2005; Kotopoulou et al. 2022). The sediment is highly enriched in Fe in the poorly crystalline fraction (51% over total), with all of them in the form of reduced Fe(II). Therefore, the formation of iron sulfides could act as a long‐term sink for iron and sulfur species.

For Mn, Phreeqc modeling did not predict the formation of MnS due to its high solubility constant. Intriguingly, there was a trend of decreasing Mn with depth in the monimolimnion, from 54 μM just below the chemocline to 41 μM at the deepest point sampled. Phreeqc modeling suggested the formation of rhodochrosite (MnCO_3_) and kutnahorite (CaMn(CO_3_)_2_) (Table S3). Manganese has been found to be closely associated with carbonates in sediments of stratified water bodies (Kiratli and Ergin 1996; Herndon et al. 2018), and rhodochrosite and kutnahorite are considered important Mn deposits in the Archean (Liu et al. 2020). Bicarbonate could be supplied in the monimolimnion by OM degradation and calcite dissolution (see Section 4.2: “Current Biogeochemical Cycling of Nutrients and Metals”). Hence, burial of Mn carbonates is a potential pathway for long‐term Mn removal, and investigation of this process in Tasik Biru could yield insights into the debated importance of Mn carbonates in the Archean (Liu et al. 2020; Lyons et al. 2020). We also note that Phreeqc modeling suggested the formation of MnHPO_4_ in the monimolimnion (Table S3). However, this assumed a maximum value of 1700 nM for phosphate based on the detection limit of the colorimetric assay. More sensitive phosphate analysis is required to determine if Mn removal as phosphate minerals is important in this lake.

In contrast to Fe and Mn, the concentrations of As were nearly identical between the two datasets spanning 20 years (Figure 5e). This implies that changes in the redox cycles of Fe, Mn, and S did not affect the net As release. The concentration of As in the surface water was ~8 μM, which is higher than the World Health Organization's recommended limit for drinking water (0.13 μM or 10 μg/L; weblink) and for the USA Environmental Protection Agency's limit for aquatic life (2–4.5 μM or 150–340 μg/L; weblink).

In conclusion, Tasik Biru is not at steady state and displays decreasing levels of Fe and Mn with increasing pH over a 20‐year period. Meanwhile, sulfate is still abundant and has the potential to be reduced to more sulfide by SRM. We predict that Tasik Biru will continue to evolve from Fe/Mn‐rich to a sulfide‐rich lake in the future, mirroring the biogeochemical transition of the Earth's ocean from the metal‐rich Archean to the Proterozoic with variable extents of sulfidic water bodies.

### 4.2 Current Biogeochemical Cycling of Nutrients and Metals

**FIGURE 8:**
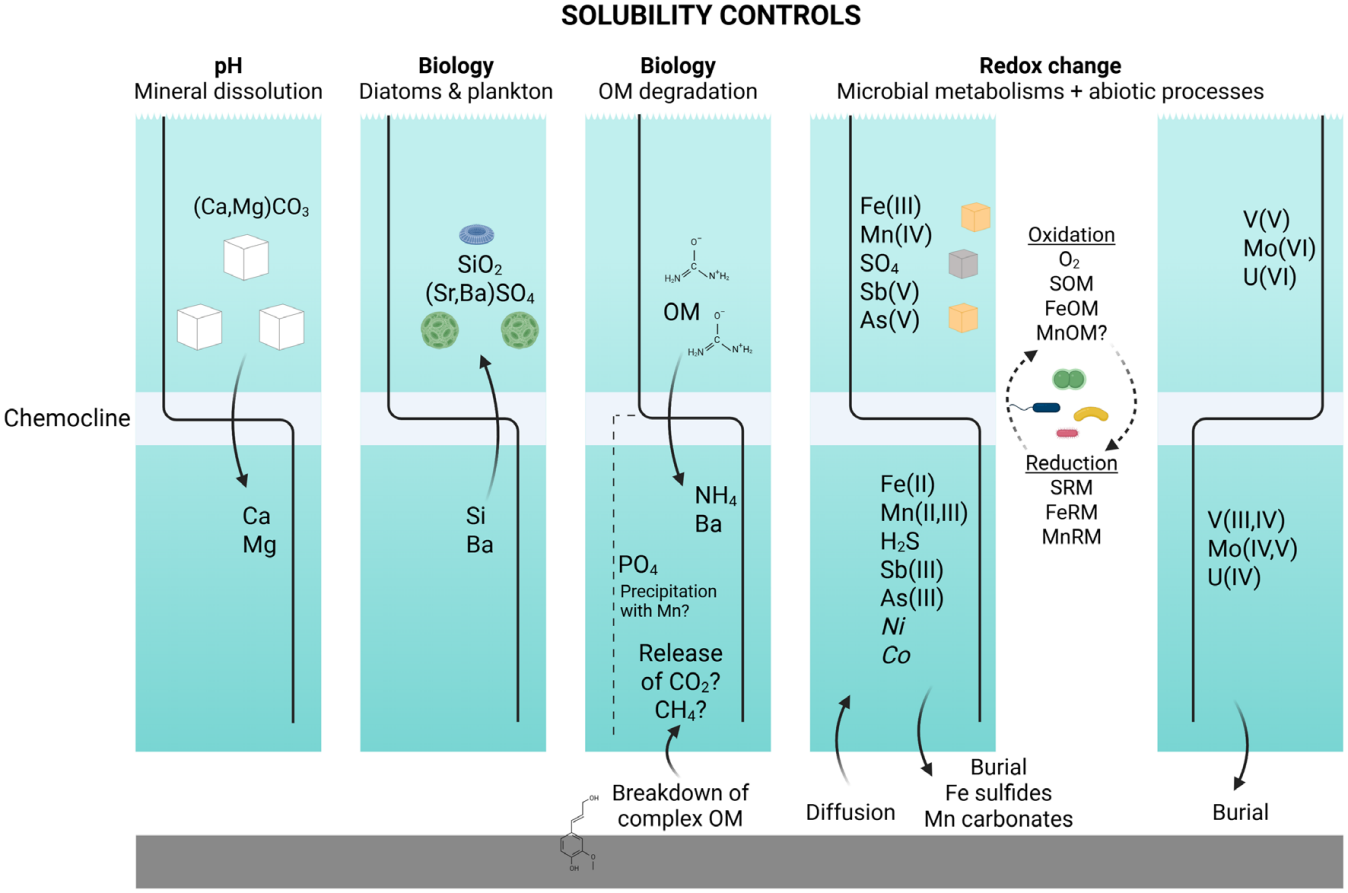
A summary of the factors affecting the depth distribution of the different elements in Tasik Biru. FeOM, iron(II)‐oxidizing microorganisms; FeRM, Fe(III)‐reducing microorganisms; MnOM, manganese‐oxidizing microorganisms; MnRM, manganese‐reducing microorganisms; SOM, sulfur‐oxidizing microorganisms; SRM, sulfate‐reducing microorganisms. Created with BioRender.com.

In this section, we present an overview of the biogeochemical cycling of nutrients and metals currently in operation, which may serve as an initial dataset for comparison to ancient ocean environments and to guide future studies as the lake is evolving (Figure 8). The microbial community distribution largely fits with the oxygen distribution, in which the mixolimnion was dominated by aerobic heterotrophs and the monimolimnion and the sediment were dominated by anaerobic carbon fermenters and sulfate‐reducing microorganisms. A majority of the elements showed an increase below the chemocline. These elements could originate from the sediments, leading to an overall increase in their concentrations in the anoxic bottom water due to the lack of physical mixing between the monimolimnion and the mixolimnion. Other reasons involving mineral precipitation and (redox) chemistry must also be considered.

For Ca and Mg, their increase in the monimolimnion may be explained as a consequence of decreasing pH, which increased the solubility of common host minerals (e.g., Mg‐containing CaCO_3_) (Figure 8). This is consistent with Phreeqc modeling that showed a slight increase in undersaturation of calcite (Table S3). For Si, low concentrations in the mixolimnion are typically explained by uptake by diatoms, which require Si to create their silica shells. Similarly, Ba can be taken up by plankton, either intracellularly or for the formation of Ba‐containing SrSO_4_ shells (Tribovillard et al. 2006; Schoepfer et al. 2015) (Figure 8).

Below the chemocline, organic matter (OM) is microbially degraded, leading to the release of NH_4_^+^ and Ba (Figure 8). Phosphate, another OM degradation product, is interestingly lower in the monimolimnion than in the mixolimnion, possibly due to precipitation with Mn (see Section 4.1: “Long‐Term Stratification and Chemical Evolution of Tasik Biru”). Organic matter degradation is coupled to microbial reduction of O_2_, sulfate, Mn(IV), and Fe(III), leading to the development of anoxic conditions and the biological production of dissolved sulfide, Mn(II), and Fe(II). Based on abundances, SRM such as *Desulfobacca* and Desulfatiglandaceae are especially important for OM degradation in the chemocline and the monimolimnion.

Surprisingly, despite the abundance of SRM, sulfate was not fully consumed, with ~75% still remaining in the monimolimnion. One explanation could be that the produced sulfide is re‐oxidized to sulfate/elemental sulfur by SOM, or is oxidized abiotically by O_2_, Fe(III), and Mn. The reaction between Fe(III) and sulfide specifically generates elemental sulfur, which then requires SOM activities for further oxidation to sulfate (Poulton et al. 2004; Jørgensen et al. 2019; Avetisyan et al. 2021). More likely, however, the sulfate profile reflects the non‐steady‐state conditions that still exist in this relatively young meromictic lake. Depleting sulfate by sulfate reduction following the establishment of persistently anoxic conditions takes time. In the 60‐m deep monimolimnion of the meromictic ferruginous Lake Towuti (Indonesia), for example, model simulations (Pu 2023; Pu et al. 2025) suggested time scales that ranged from several months (after weak ventilation of the monimolimnion with oxygen‐bearing water) to several decades (after full oxygenation). In either scenario, sulfate was still not fully consumed in that time. Such features are also observed in Lake Medard (Czech Republic), a post‐mining lake less than 16 years old that is rich in elevated sulfate alongside ferruginous bottom water (Petrash et al. 2022). A secondary groundwater source of sulfate in Tasik Biru also cannot be ruled out.

An interesting observation is the lack of DOC buildup in the monimolimnion despite evidence of OM degradation. This suggests that a major portion of C‐derived OM was converted to inorganic carbon (e.g., dissolved HCO_3_^−^) or CH_4_. The production of CH_4_ is likely given the enrichment of *Methylomirabilis* at the chemocline. As mentioned previously, this genus is known for catalyzing nitrite‐dependent methane oxidation. Other methane‐oxidizing bacteria such as *Methylomonadaceae*, *Methylocystis*, and *Methylococcaceae* are present at low abundances. Surprisingly, known methanogens such as Methanomicrobia, Methanosarcina, and Methanomassiliicoccales are only present at low abundances (< 0.5%) in Tasik Biru, despite the relatively high level of Ni and the presence of complex OM degraders in the sediment that could contribute to prime methanogenesis. The balance between CH_4_ production and consumption is important to understand in both the modern and ancient Earth, given the ability of CH_4_ to act as a greenhouse gas. Hence, future research to identify methanogenic archaea and the fate of methane as a function of C, Fe, S, and Mn availability is warranted.

The increasing concentrations of Fe and Mn below the chemocline are often explained by a tight cycling between their redox states (Tribovillard et al. 2006). Their dissolved depth profiles suggest that the two elements are diffusing upwards from the sediment into the O_2_‐rich region, where they could be oxidized abiotically by O_2_ or via the activity of FeOM. MnOM was not detected based on our microbial community analysis, but we could not rule out the involvement of other unknown microbial taxa. Phreeqc modeling (Table S3) predicted the formation of ferrihydrite (FeOOH), jarosite ([KFe_3_(SO_4_)_2_(OH)_6_]), magnetite (Fe_3_O_4_), and strengite (FePO_4_٠2H_2_O) where dissolved Fe^3+^ was detected. The presence of these minerals could explain the detection of “dissolved” Fe(III) in the water column if the particle size is small enough to pass through a 0.22 μm filter. Some Fe(III) could also be stabilized in the aqueous phase via complexation with natural OM (Lead and Wilkinson 2006; Hochella et al. 2019).

Upon settling back below the chemocline, the formed Fe(III) and Mn(IV) minerals would be re‐reduced by the activity of FeRM and MnRM or abiotically by reaction with sulfide and reduced OM. The zone of Mn release was slightly shallower than for Fe (Figure 4c,d). It is likely that reactive Mn(IV) oxides are acting as oxidants for dissolved Fe^2+^ upon sinking, leading to an apparent zone of Mn dissolution above the zone of Fe^2+^ release. This is also observed in other stratified lakes, consistent with the relative oxidation kinetics and redox potentials of Mn compared to Fe (Havig et al. 2015; Janssen et al. 2024). However, the role of MnOM and MnRM in mediating direct Mn redox transformation is relatively unknown. Given the unusual richness of Mn relative to Fe in Tasik Biru (~1:1 ratio), this site may be particularly intriguing for investigation on Mn biogeochemical cycling.

Keeping in mind the non‐steady‐state condition, we also conducted Phreeqc modeling using the pH, dissolved Fe, Mn, and As dataset of Sari et al. (2006) while keeping all other parameters constant to investigate if mineral precipitation could have changed over time. This model predicted generally more negative saturation states of Fe(II) minerals, calcite, rhodochrosite, and kutnahorite due to the lower pH in the monimolimnion, and no large changes in the saturation state of quartz and barite compared to our study. In‐depth characterization of minerals in the water column and the sediments is needed to better link water column geochemistry to proxy preservation over long time scales.

Other elements such as Sb, As, Ni, Co, V, Mo, and U also showed depth‐dependent behavior most easily explained due to differences in solubility as a function of redox conditions (Tribovillard et al. 2006). Arsenic increased below the chemocline due to the predominance of the reduced and more soluble As(III) compared to the oxidized and less soluble As(V) (Figure 8d). In contrast, Sb, V, Mo, and U decreased below the chemocline due to the predominance of the reduced and less soluble Sb(III), V(III, IV), Mo(IV, V), and U(IV) compared to the oxidized and more soluble Sb(V), V(V), Mo(VI), and U(VI) (Figure 8e). Microorganisms likely play a role in these redox reactions (Gustafsson 2019; Phillips and Xu 2021; Yin et al. 2022; Fu et al. 2023; Qiuhan and Ouyang 2023; William and Magpantay 2024). Molybdenum may also precipitate as Mo‐sulfides (Table S3). Furthermore, while Ni and Co are not directly redox‐active, their behavior is often controlled by association to Mn(IV) and Fe(III) minerals that are being reduced at and below the chemocline. Their rapid increase in the zone of Mn and Fe release, followed by a decrease to lower values deeper in the monimolimnion (Figure 5h,i), could be explained by the formation of Co and Ni‐sulfides (Table S3). These would also explain their enrichment in the sediment (EF = 3.2–7.1), particularly in the poorly crystalline and HCl‐extractable fractions (Figure 6, Table S2).

In summary, many open questions remained including on (a) an additional sulfate source in the monimolimnion, (b) the level of methane production and its microbial origin, (c) the relative contribution of biotic versus abiotic Mn redox transformation, and (d) linking water column to solid phase geochemistry for proxy preservation.

### 4.3 Tasik Biru's Comparison to Ancient Oceans and Modern Stratified Lakes

Over a large span of geologic time, variations in the mixing dynamics and geochemistry (e.g., O_2_ level, nutrients, and metal contents) in the ancient oceans created combinations of physical and chemical conditions that encompassed a wide range. For example, the early Archean ocean (4–2.5 Ga) was likely rich in iron (40–120 μM) and manganese (~10 μM) and low in O_2_ (< 0.2 μM) (Table 1). The evolution of oxygenic photosynthesis likely led to the formation of oxygen oases in shallow waters (up to tens of μM) in the Neoarchean (3.0–2.5 Ga). As O_2_ rose in the atmosphere, overlapping zones of reduced species and dissolved O_2_ would have expanded, generating zones including water column chemoclines with an expanded aerobic biosphere (Runge et al. 2025). Continued O_2_ build‐up in the atmosphere would have accelerated pyrite weathering on land, resulting in sulfate input to the ocean with estimated concentration rising to up to 2000 μM (Fakhraee et al. 2025). The activity of sulfate‐reducing microorganisms would then have been favored, leading to the expansion of localized sulfidic zones in the Proterozoic ocean (Lyons et al. 2024). Despite increased sulfate input, the deep Proterozoic ocean was likely still ferruginous, with expanding occurrences of sulfidic margins from the Paleoproterozoic (2.5–2.0 Ga) to the mid‐Proterozoic (2.0–1.0 Ga) (Lyons et al. 2024; Fakhraee et al. 2025). Water columns containing reduced Fe^2+^, Mn, and sulfide would become rarer as O_2_ concentration builds up to modern levels, as observed today. Thus, the stability of Tasik Biru's stratification and its biogeochemical cycling would dictate its comparison to the most relevant time period in Earth's history.

**TABLE 1:** Comparison of geochemical parameters in the monimolimnion of Tasik Biru with oceans throughout Earth's history.

Units	Parameter	Archean ocean^a^	*Tasik Biru monimolimnion*	Proterozoic ocean^b^	Modern ocean^c^
	pH	> 6.5	6.9	> 6.5	8.1
μM	O_2_	< 0.2	12‐30^d^	24–120	325
μM	Fe(II)	40–120	23–54	0.1–0.5	< 0.001
μM	Mn	10	39–54		< 0.002
μM	Sulfide		< 4		< 0.001
μM	Sulfate	5–200	255	500–3000	28,000
μM	Si	670–2200	152–186	670–2200	10–180
μM	Ca	10,000	1350–1630		10,000
μM	K		21–23		10,000
μM	Mg	100,000	125–157		53,000
μM	Na		82–83		469,000
μM	As		20–22		
nM	PO_4_	40–130	< 1700	40–130	170–1440
nM	Sb		6–12		
nM	V	0.001	4–5		40
nM	Ba		313–378		109
nM	Ni	1–10	11–26	1	10
nM	Co	10	54–107	0.1	0.02
nM	Mo	1	2–9	10	100
nM	U	0.01–0.1	0.4–0.6	0	13
nM	Se		< 62		
nM	Cu	10^−13^	< 3	10^−16^	2.36
nM	Zn	< 10	< 18	< 10	5

*Note:* References: Tribovillard et al. (2006); Anbar (2008); Konhauser et al. (2015); Koeksoy et al. (2016); Robbins et al. (2016).

^a^ Ferruginous end member.

^b^ Euxinic end member.

^c^ Oxic end member.

^d^ Likely higher than real value due to atmospheric equilibrium during analysis.

The current monimolimnion of Tasik Biru could be summarized as Fe‐ and Mn‐rich, with moderate sulfate levels and with relatively low sulfide and Si. Table 1 compares the geochemical parameters of the lake's monimolimnion to those in the ocean in different periods of Earth's history. Its aqueous geochemistry straddles the geochemical ranges between the Archean and the Proterozoic, especially for the concentrations of O_2_, Fe, sulfate, Mo, and U. Hence, Tasik Biru is currently most similar to a Neoarchean ocean (3.0–2.5 Ga) that was receiving sulfate input and transitioning to the Paleoproterozoic (2.5–2.0 Ga) with higher influence of sulfide. Over time, the lake may slowly transition to a more sulfide‐rich water column, becoming more similar to conditions in the mid‐Proterozoic (2.0–1.0 Ga). We propose that Tasik Biru could join a list of modern stratified lakes with ferruginous or sulfidic monimolimnions that have been studied as analogues of the ancient oceans. Continuous monitoring over time of shifts in microbial communities, water column geochemistry, and sedimentary compositions of this lake may provide unique insights into this momentous period in Earth's history.

Various modern stratified lakes with ferruginous or sulfidic monimolimnions have been proposed to serve as model systems for the Precambrian oceans (Hamilton et al. 2016; Koeksoy et al. 2016; Swanner et al. 2020). Comparison of Tasik Biru to a select few of these lakes [Brownie Lake (USA), Canyon Lake (USA), Lake Pavin (France), Lake Matano (Indonesia), Lake Poso (Indonesia), Fayetteville Green Lake (USA)] (Balistrieri et al. 1994; Michard et al. 1994; Viollier et al. 1995; Crowe et al. 2008; Havig et al. 2015; Lambrecht et al. 2018; Cole et al. 2020) highlights that it is most geochemically similar to Lake Poso, an ancient 350 m deep lake in Indonesia (Janssen et al. 2024), albeit with generally 2–3× higher elemental concentrations (Table S4). This reflects local geological controls of Tasik Biru's water column chemistry based on leaching of the residual economic deposits rich in Fe, Mn, As, Sb, and other metals. Disentangling local controls from universal geochemical features of stratified lakes is an important question to guide the selection of model systems for the ancient oceans (Janssen et al. 2024). Investigation of Tasik Biru offers one key advantage, in that it is not in steady‐state. Therefore, dynamic biogeochemical changes can be studied in more detail. Furthermore, it is intriguing to note that many of the lakes that were studied previously contain incomplete trace metal datasets. Since trace metals are critical drivers of biogeochemical evolution due to their participation in enzymes and metabolisms, we encourage future studies to include trace metal analysis whenever possible (Glass and Orphan 2012; Moore et al. 2017).

The geochemistry of Tasik Biru differs from the ancient oceans in several important respects (Table 1). First, Si concentration in Tasik Biru is about 5× lower than the minimum estimate in the ancient oceans. The presence of Si played an important role in stimulating primary Fe‐Si mineral precipitates that formed in the Archean ocean, with important implications for reading the geological record of Banded Iron Formations (Dreher et al. 2021, 2025). Second, the phosphate concentration in the monimolimnion of Tasik Biru (< 1700 nM) was near the detection limit of the colorimetric assay but still at least 13× higher than the estimated levels in the ancient oceans (40–130 nM) for the Archean and Proterozoic oceans; (Koeksoy et al. 2016). Exact concentrations could make a difference for the biogeochemical cycling of Fe, Mn, and Ca as they determine the relative precipitation of vivianite, Mn phosphates, and apatite (Ca‐phosphates). Finally, the concentrations of Ni (2–20×) and Co (5–1000×) in the lake are higher than in the ancient oceans. Ni plays an important role as the active enzyme center responsible for methane production by methanogenic archaea. The drop in Ni availability in the Archean was invoked in the “nickel famine” hypothesis, in which methane production was retarded, leading to less greenhouse gases and subsequent adaptation and evolution of microbial life to the new atmosphere (Konhauser et al. 2015). Meanwhile, Co is essentially needed for vitamin B12 biosynthesis and also participates in carbon compound transformation involved along the methanogenesis pathway (Glass and Orphan 2012; Moore et al. 2017). Hence, these differences need to be considered when making interpretations of ancient oceans based on Tasik Biru.

## Conflicts of Interest

The authors declare no conflicts of interest.

## Supporting information


**Figure S1:** (a) Color of the lake water after ~30 min of collection from Tasik Biru. All water samples were clear at the beginning. Once exposed to air, water samples from 57.5 m and 65 m turned orange within 30 min, indicative of Fe^2+^ oxidation and precipitation as Fe(III) (oxyhydr)oxide minerals. (b) Water column particulates from the mixolimnion that were collected on the filter after exposure to O_2_. (c) Water column particulates from the monimolimnion after exposure to O_2_. Note that the orange color from the Fe(III) minerals is due to oxidation during sampling, and thus does not represent their actual presence at depth.
**Figure S2:** Results of XRD analysis of samples from Tasik Biru. Both the soil (from the shore) and sediment (at 58 m water depth) samples show signals of quartz and a kaolinite‐like clay phase. Reference patterns are shown in gray at the bottom. Quartz: PDF 96‐901‐3322. Kaolinite: PDF 96‐901‐3322.
**Figure S3:** Rarefaction curves comparing the alpha diversity (Shannon's diversity index) in the water column (depths indicated) and sediments (Sed) of Tasik Biru (a) sequenced with bacterial‐specific primers and (b) sequenced using universal primers, marked with an asterisk.
**Figure S4:** Sankey diagrams of microbial communities in the lake. The plot was created using the web version of Pavian (https://shiny.hiplot.cn/pavian/) (Breitwieser and Salzberg 2020) with only the five most abundant taxa shown at each taxonomic level. Asterisk (*) after the sample name indicates sequencing using universal primers, while the rest was sequenced using bacterial‐specific primers. Numbers above each node indicate the relative abundances.
**Figure S5:** Abundances of archaeal ASVs relative to total archaea in the water column (depths indicated) and sediment (Sed) of Tasik Biru. The percentages of total archeaea are shown at the top.
**Figure S6:** FAPROTAX functional prediction based on microbial community data of water column (depths indicated) and sediment (Sed) of Tasik Biru. Samples marked with an asterisk (*) indicate sequencing via universal primers, while the rest were sequenced with bacterial‐specific primers.
**Table S1:** Detection limits for all analysis.
**Table S2:** Solid‐phase contents of sediment (58 m water column depth) and soil (near the shore) from Tasik Biru.
**Table S3:** Saturation (SI) of minerals across water column depth as determined via Phreeqc modeling. Blue is undersaturated while red is oversaturated, with the color gradient indicative of the degree of saturation.
**Table S4:** Comparison of geochemical parameters in the monimolimnion of Tasik Biru to other modern stratified lakes. The lakes are arranged from Fe‐rich (left) to sulfide‐rich (right).

## Data Availability

DNA sequences have been deposited in the NCBI Short Read Archive under the BioProject ID PRJNA1223325. All other data are available in this manuscript.
