# Supplementary material for: Biogeochemistry of an Iron‐ and Manganese‐Rich Stratified Lake: Tasik Biru, Malaysia, as a Modern Model Habitat for the Ancient Ocean
Source: Geobiology. 2025 Oct 11;23(5):e70036. doi: 10.1111/gbi.70036 (PMC12515063; doi:10.1111/gbi.70036)
Supplement: Supplementary file 1 — Figure S1: (a) Color of the lake water after ~30 min of collection from Tasik Biru. All water samples were clear at the beginning. Once exposed to air, water samples from 57.5 m and 65 m turned orange within 30 min, indicative of Fe2+ oxidation and precipitation as Fe(III) (oxyhydr)oxide minerals. (b) Water column particulates from the mixolimnion that were collected on the filter after exposure to O2. (c) Water column particulates from the monimolimnion after exposure to O2. Note that the orange color from the Fe(III) minerals is due to oxidation during sampling, and thus does not represent their actual presence at depth. Figure S2: Results of XRD analysis of samples from Tasik Biru. Both the soil (from the shore) and sediment (at 58 m water depth) samples show signals of quartz and a kaolinite‐like clay phase. Reference patterns are shown in gray at the bottom. Quartz: PDF 96‐901‐3322. Kaolinite: PDF 96‐901‐3322. Figure S3: Rarefaction curves comparing the alpha diversity (Shannon's diversity index) in the water column (depths indicated) and sediments (Sed) of Tasik Biru (a) sequenced with bacterial‐specific primers and (b) sequenced using universal primers, marked with an asterisk. Figure S4: Sankey diagrams of microbial communities in the lake. The plot was created using the web version of Pavian (https://shiny.hiplot.cn/pavian/) (Breitwieser and Salzberg 2020) with only the five most abundant taxa shown at each taxonomic level. Asterisk (*) after the sample name indicates sequencing using universal primers, while the rest was sequenced using bacterial‐specific primers. Numbers above each node indicate the relative abundances. Figure S5: Abundances of archaeal ASVs relative to total archaea in the water column (depths indicated) and sediment (Sed) of Tasik Biru. The percentages of total archeaea are shown at the top. Figure S6: FAPROTAX functional prediction based on microbial community data of water column (depths indicated) and sediment (Sed) of Tasik Biru. Sa [file GBI-23-e70036-s001.docx]

**Supporting information**

**Biogeochemistry of an iron- and manganese-rich stratified lake: Tasik Biru, Malaysia, as a modern model habitat for the ancient ocean**

**
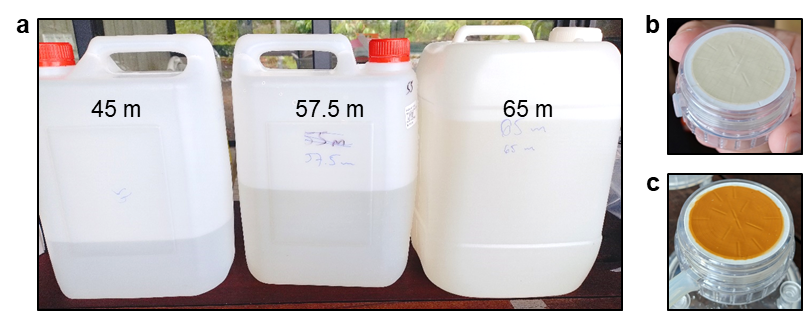
**

**Figure S1:** (a) Color of the lake water after ~30 minutes of collection from Tasik Biru. All water samples were clear at the beginning. Once exposed to air, water samples from 57.5 m and 65 m turned orange within 30 minutes, indicative of Fe^2+^ oxidation and precipitation as Fe(III) (oxyhydr)oxide minerals. (b) Water column particulates from the mixolimnion that were collected on the filter after exposure to O_2_. (c) Water column particulates from the monimolimnion after exposure to O_2_. Note that the orange color from the Fe(III) minerals is due to oxidation during sampling, and thus does not represent their actual presence at depth.


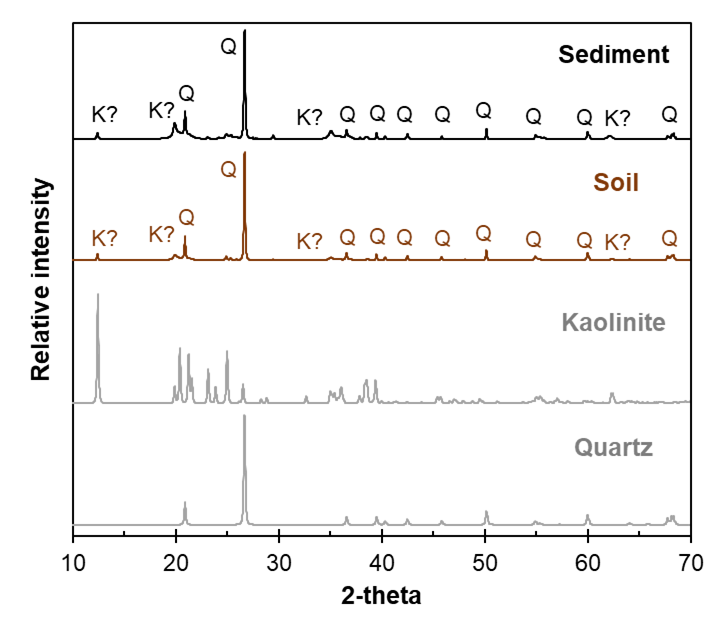


**Figure S2:** Results of XRD analysis of samples from Tasik Biru. Both the soil (from the shore) and sediment (at 58 m water depth) samples show signals of quartz and a kaolinite-like clay phase. Reference patterns are shown in gray at the bottom. Quartz: PDF 96-901-3322. Kaolinite: PDF 96-901-3322.

**
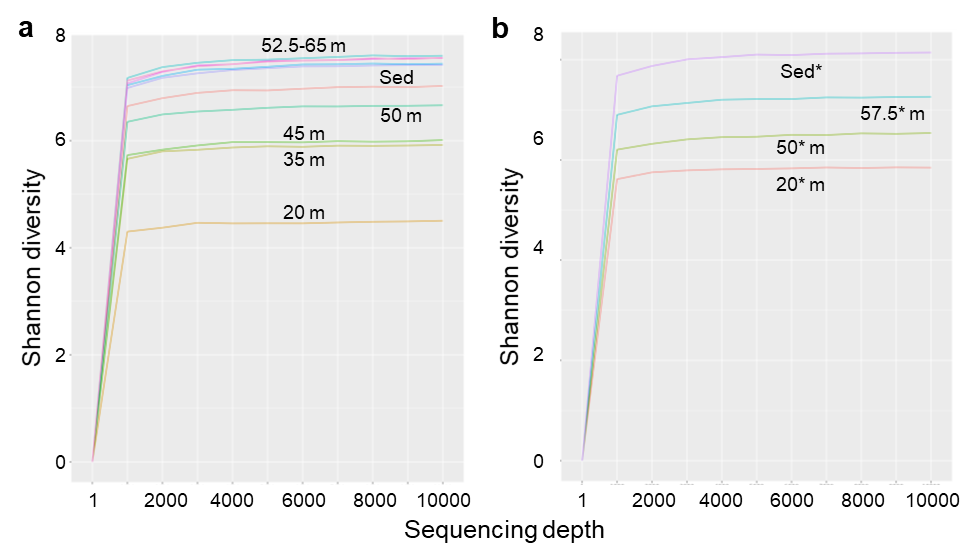
**

**Figure S3**: Rarefaction curves comparing the alpha diversity (Shannon’s diversity index) in the water column (depths indicated) and sediments (Sed) of Tasik Biru (a) sequenced with bacterial-specific primers and (b) sequenced using universal primers, marked with an asterisk.

**Appendix S1: In-depth discussion of bacterial community.**

DNA extraction from water samples resulted in yields of 198-540 ng DNA/L of water, which corresponded to 1.8-5.4 x 10^5^ cells/ml in the water column (assuming 1 fg DNA per cell based on *Escherichia coli*). These values are lower than the estimated average of 106 cells/ml for lakes globally (Whitman et al., 1998). Extraction from the lake sediment yielded 1188 ng DNA/g wet sediment. This corresponded to 1.6 x 109 cells/cm3 (assuming a porosity of 0.8 and an average solid phase density of 2.7 g/cm^3^), which is higher than the estimated global average of 0.5 x 10^9^ cells/cm^3^ (Whitman et al., 1998). 16S rRNA amplicon sequencing yielded sufficient reads that capture the microbial diversity, as indicated by rarefaction curves that plateau with increasing sequencing depth (**Fig. S3**).

In the mixolimnion, the bacterial community was dominated by the order Pedosphaerales (16-47%), the family SM1A02 (6-14%), and the genera BACL27 (2-6%), Hypericibacter (5-8%) and Gaiella (0.7-6%). The order Pedosphaerales is widespread in soil and is known to be preferentially associated with large macroaggregates (Bach *et al.*, 2018). The family SM1A02 have been found in sludge, wastewater treatment bioreactors and in association with microalgae (Tian *et al.*, 2017; Rambo *et al.*, 2020; Vico *et al.*, 2021), where their role in anaerobic ammonium oxidation (anammox) has been postulated but not confirmed (Tian *et al.*, 2017; Wang *et al.*, 2023). The BAltic Sea CLusters (BACL; Sjöqvist et al., 2021) belong in the Ilumatobacteraceae family, for which the type strain is an aerobic heterotroph (Asem *et al.*, 2018). The genus *Hypericibacter* can grow under oxic or microoxic conditions with a range of carbon substrates (Noviana *et al.*, 2020). The genus *Gaiella* is also an aerobic heterotroph, and growth was not observed under anoxic conditions (Albuquerque *et al.*, 2011; Severino *et al.*, 2019).

The monimolimnion bacterial community was dominated by the genus *Desulfobacca* (3-13%), the family EnvOPS12 (5-8%), UBA12465 (1-6%) and Desulfatiglandaceae (3-13%), and the order XYA2-FULL-43-10 (2-5%). *Desulfobacca* and Deulfatiglandaceae are both sulfate reducers but they utilize different organic substrates i.e., only acetate for *Desulfobacca* and various simple and complex organic substrates (including aromatics) for Desulfatiglandaceae (Trujillo *et al.*, 2015). The family EnvOPS12 has been found in a number of anoxic environments (Yamashita *et al.*, 2019) but their metabolism is poorly known, as genomic assemblies are challenging due to the high diversity (Singleton *et al.*, 2021). They belong to the Anaerolinaea class that are known to be filamentous anaerobic complex carbon degraders (Suominen *et al.*, 2021). The family UBA12465 (UBA = Uncultivated Bacteria and Archaea; Parks et al., 2017) and XYA2-FULL-43-10 belongs to the Candidate Phyla Radiation (CPR, also known as the Patescibacteria phylum). Bacteria in this phylum are typically very small, with reduced genome sizes, simple membranes, and potential symbiotic relationship with other microorganisms (Tian *et al.*, 2020; Kagemasa *et al.*, 2022; Jaffe & Banfield, 2024).

The sediment bacterial community was dominated by the family Lachnospiraceae (28%) and the genera *Faecalibacterium* (11%), *Blautia_A* (9%), *Bifidobacterium* (4%) and 0-14-3-00-41-53 (3%). The first three are all located within the class Clostridia (44% over total bacteria) and are known as anaerobic fermenters of complex organic carbon, typically encountered in human intestines and fecal samples (Duncan *et al.*, 2002; Cotta & Forster, 2006; Miura *et al.*, 2023). Likewise, *Bifidobacterium* within the class Actinomyceta is known as anaerobic fermenter associated with the mammalian gut (Turroni *et al.*, 2011). The genus 0-14-3-00-41-53 is affiliated with the class Thermodesulfovibrionia, which is generally capable of sulfate reduction coupled to complete denitrification (Diao *et al.*, 2023; Mosley *et al.*, 2024).

**Appendix S2: In-depth discussion of archaeal community.**

We focus on the top archaeal ASVs down to the order level to investigate putative metabolic functions (**Fig. S5**). The mixolimnion was dominated by the Nitrososphaerales (92% over total archaea), which are aerobic ammonia-oxidizers that occur widely in marine and terrestrial ecosystems (Pester *et al.*, 2011). The ASV in Tasik Biru is related most closely to *Candidatus* Nitrosotenuis aquarius (group I.1a *Thaumarchaeota*) isolated from an aquarium biofilter that oxidize ammonia to nitrite (Sauder *et al.*, 2018). The order Nitrososphaerales was also recently implicated in sulfur oxidation based on genomic analysis (Qi *et al.*, 2024).

The monimolimnion was dominated by the Pacearchaeales (86% over total archaea). This order is a part of the DPANN superphylum (Nanoarchaeota phylum) that are ubiquitous in nature. They are associated with small genomes that encode for fermentation and occasionally CO_2_ fixation, but their limited metabolic capabilities likely imply a symbiotic lifestyle with other microorganisms (Baker *et al.*, 2020; Dopson *et al.*, 2024; Qi *et al.*, 2024). The ASV in Tasik Biru is most closely related to the recently described family GW2011-AR1 observed in deep groundwaters of the Fennoscandian Shield (Dopson *et al.*, 2024).

The chemocline exhibited more diverse archaeal community representing mixtures of the mixoliminion and the monimolimnion, comprising of Pacearchaeales (28%), Bathyarchaeales (23%), Nitrososphaerales (9%), the class Thermoplasmata (4%) and others with lower abundances (36% total). The Bathyarchaeales (phylum Thermoproteota, class Bathyarchaeia), part of the TACK superphylum, were relatively high at the chemocline compared to the waters above and below. They are involved in degradation of various complex organic matter (lignin, proteins, alkanes), are able to fix CO_2_ by the Wood-Ljungdahl pathway, and are also implicated in sulfur oxidation and H_2_-based respiration (Baker *et al.*, 2020; Qi *et al.*, 2024). A model organism named *Bathyarchaeum tardum* was recently cultivated from lake sediments. This organism grows in co-culture with methanogens and is able to anaerobically degrade complex proteinaceous substrates and methoxylated aromatic compounds such as DMB and vanillate (Khomyakova *et al.*, 2023).

Lastly, the sediment was mostly comprised of a mixture of Bathyarchaeales (69%) and the class Thermoplasmata (19%). The Bathyarchaeales sequences in Tasik Biru sediment are most closely related to the family Bathy-6 (also known as MCG-6 or UBA233), which are implicated in fermentation in sulfidic lakes, peat soils, sediments, anaerobic digesters and intestines of terrestrial arthropods (Fillol *et al.*, 2015; Protasov *et al.*, 2023). The Thermoplasmata (Euryarchaeota phylum) was originally described from oxic and acidic environments, but has since been found in freshwater sediments, fens and marine subsurface where they are implicated in the degradation of proteins and long-chain fatty acids (Compte-Port *et al.*, 2017; Baker *et al.*, 2020). Co-occurrences of Bathyarchaeales and Thermoplasmata have been described previously in various freshwater systems where they are likely involved in anaerobic carbon degradation (Compte-Port *et al.*, 2017).

**Appendix S3: Comparison between bacterial communities determined from bacterial-specific versus universal primers.**

In general, the bacterial community composition was mostly similar between the two sequencing sets, with the exception of the sediment (**Fig. S4m-n**). The reason for the discrepancy is likely due to primer biases, although we cannot rule out contamination during sequencing for this particular sample. In particular, ASVs from bacterial-specific primers were dominated by the phylum Firmicutes (44%, e.g., family Lachnospiraceae and genera *Faecalibacterium* and *Blautia_A*), Actinobacteriota (8%, e.g., genus *Bifidobacterium*) and Bacteroidota (4.4%). These phyla were largely absent (< 0.3%) in ASVs from universal primers. Other than that, the two datasets share many bacterial ASVs including those belonging to the class Thermodesulfovibrionia (3-5% e.g., genus 0-14-3-00-41-53), Dehalococcoidia (9.5-9.9%), BSN033 (2.5-5.9%) and Aminicenantia (2.3-7.7%). The class Dehalococcoidia (phylum Chloroflexi) is implicated in widespread organohalide reduction coupled to H_2_ oxidation (Yang *et al.*, 2020). The class BSN033 belongs to cluster D of the phylum Desulfobacterota, associated with sulfate reduction and anaerobic hydrocarbon degradation (Langwig *et al.*, 2022). The class Aminicenantia (phylum Acidobacteriota) is also implicated in anaerobic degradation of complex carbohydrates, fatty acids and proteins, perhaps coupled to reduction of iron (oxyhydr)oxides (Booker *et al.*, 2023). Hence, despite the discrepancies in microbial communities between the two sequencing sets, the inferred dominant metabolic capabilities for anaerobic carbon degradation and sulfate reduction remained the same, with an intriguing lack of abundance of known methanogenic archaea.


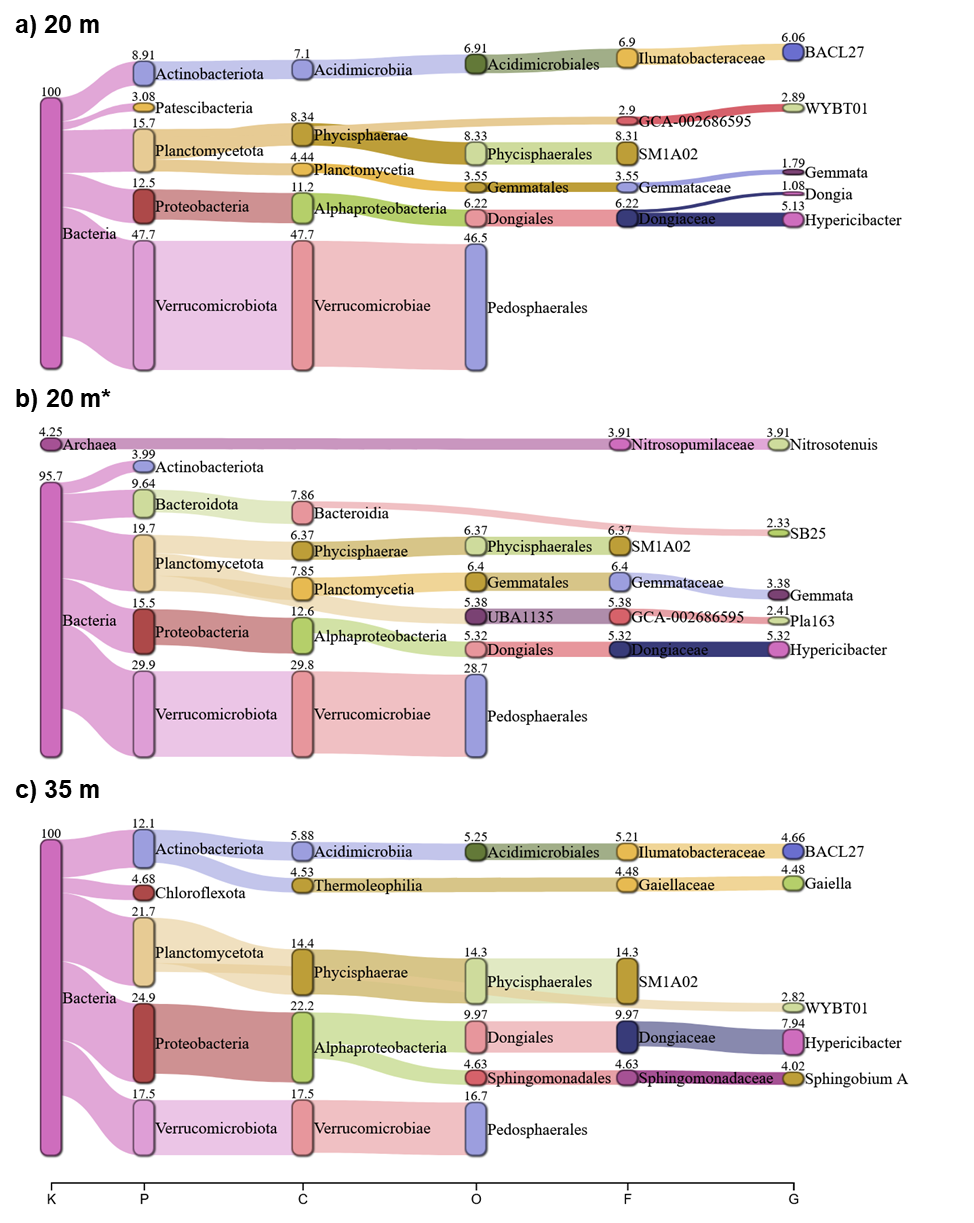


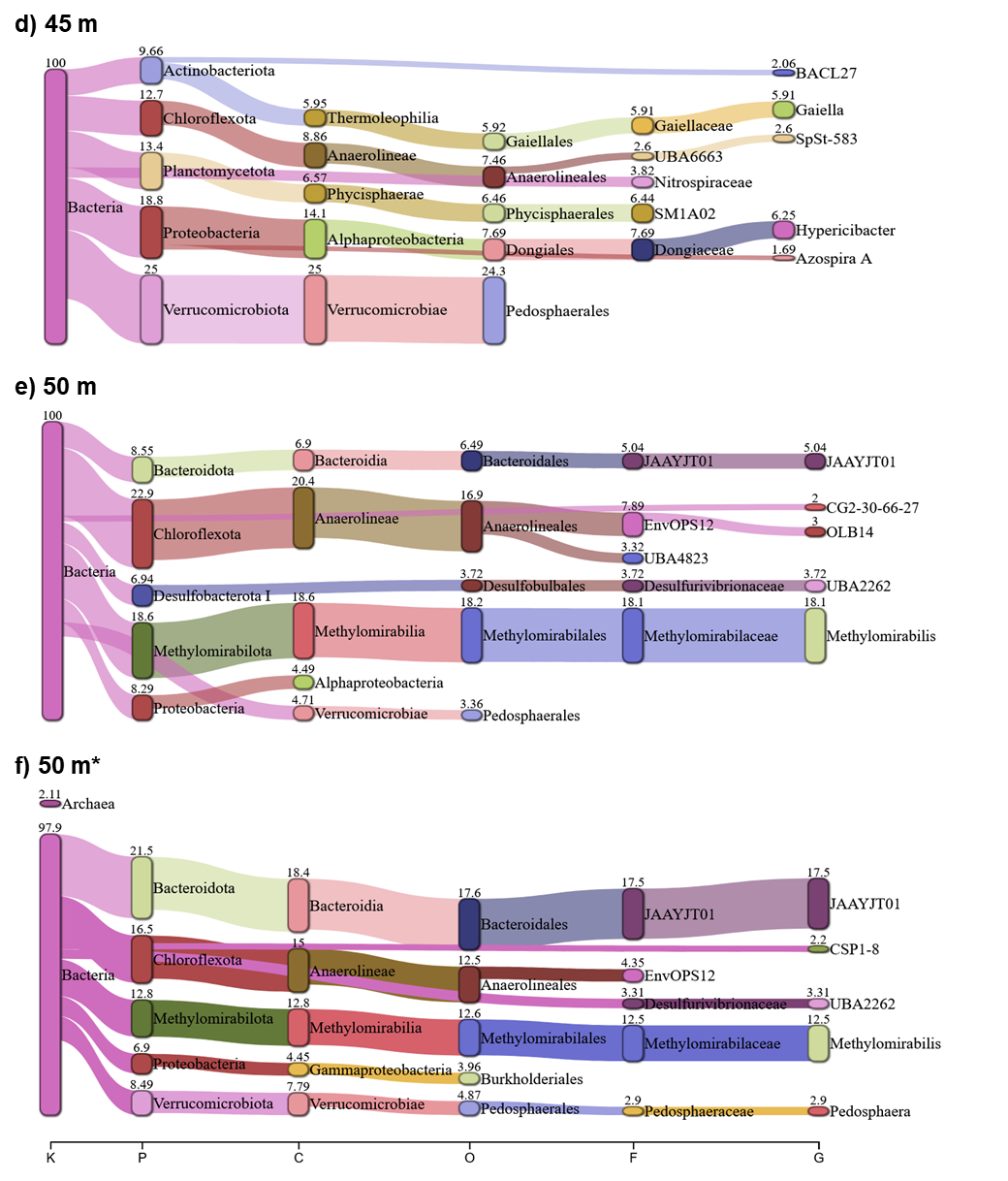


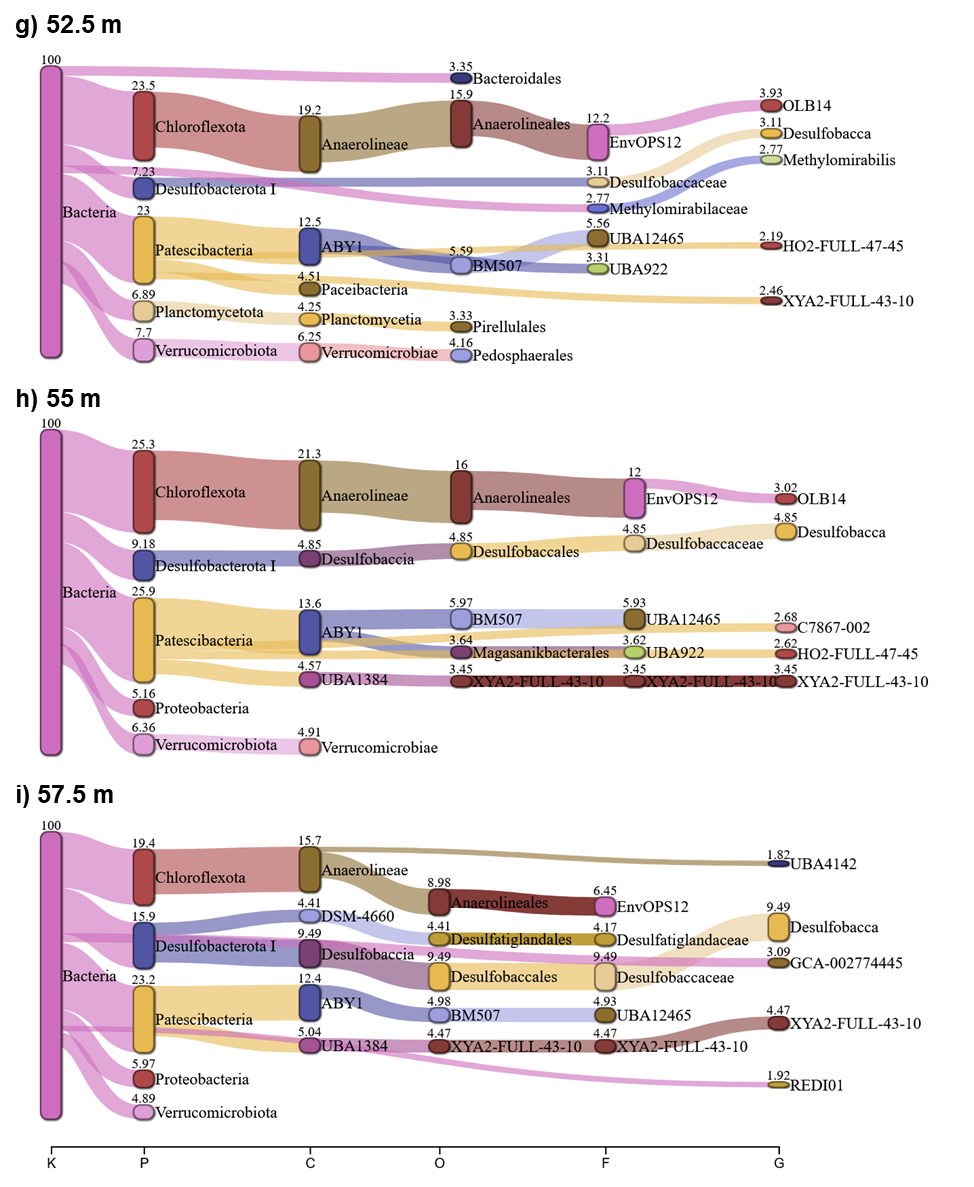


**
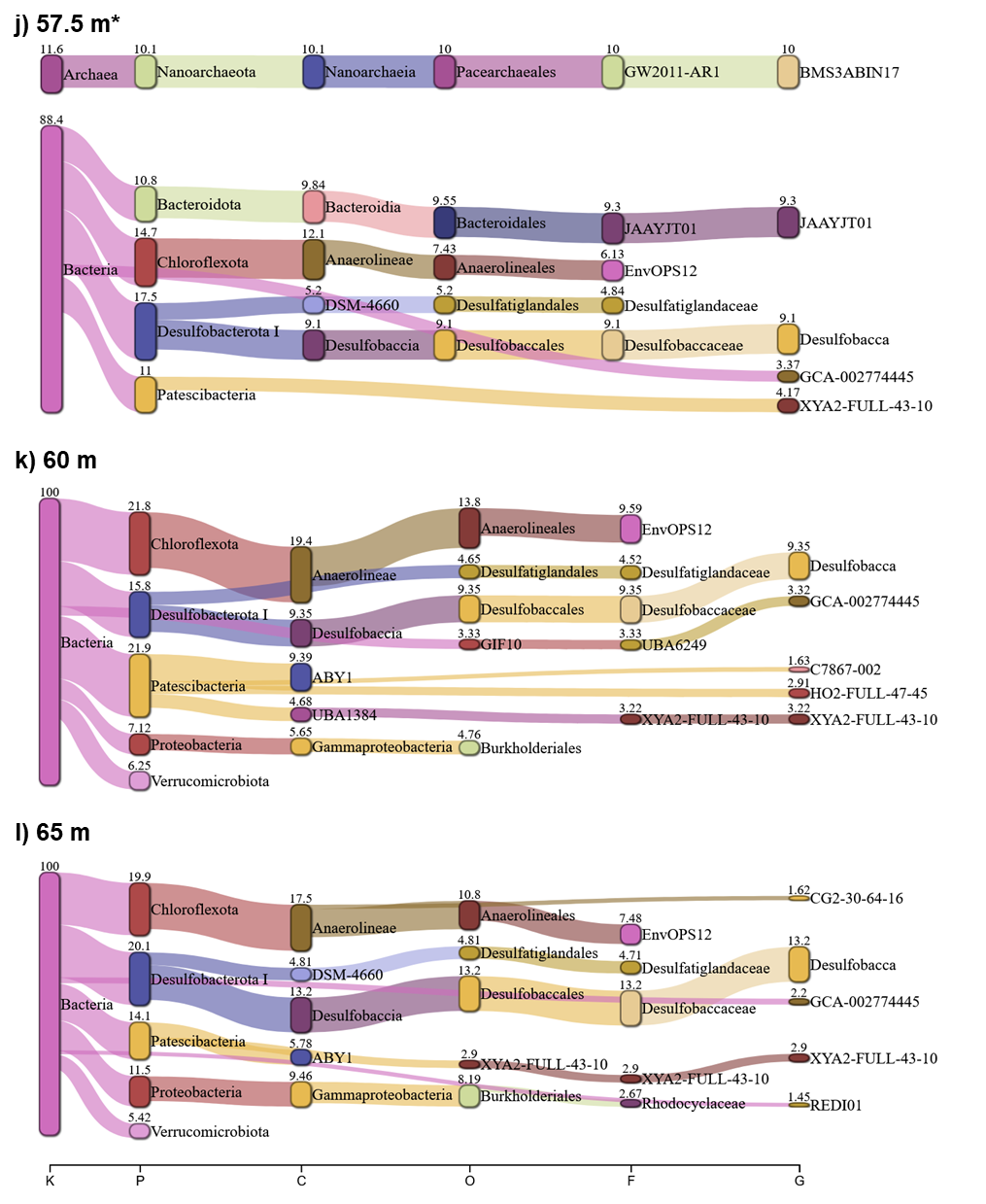
**

**
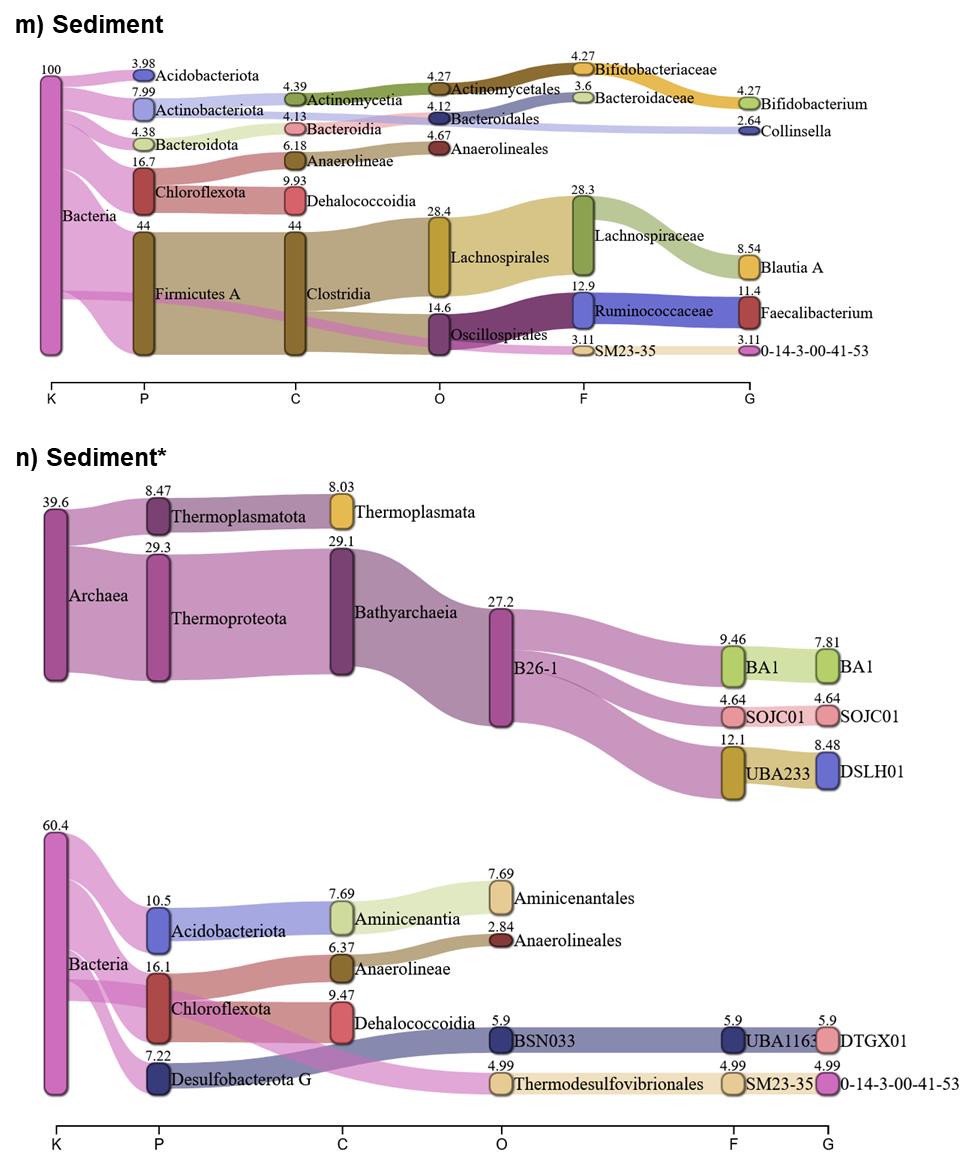
**

**Figure S4**: Sankey diagrams of microbial communities in the lake. The plot was created using the web version of Pavian (<https://shiny.hiplot.cn/pavian/>) (Breitwieser & Salzberg, 2020) with only the 5 most abundant taxa shown at each taxonomic level. Asterisk (*) after the sample name indicates sequencing using universal primers, while the rest was sequenced using bacterial-specific primers. Numbers above each node indicate the relative abundances.


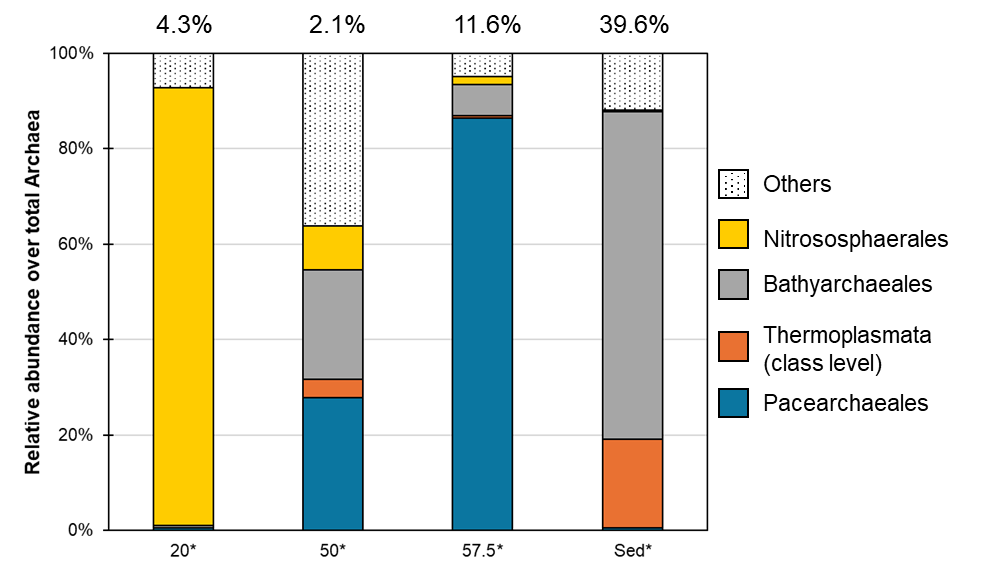


**Figure S5**: Abundances of archaeal ASVs relative to total archaea in the water column (depths indicated) and sediment (Sed) of Tasik Biru. The percentages of total archeaea are shown at the top.


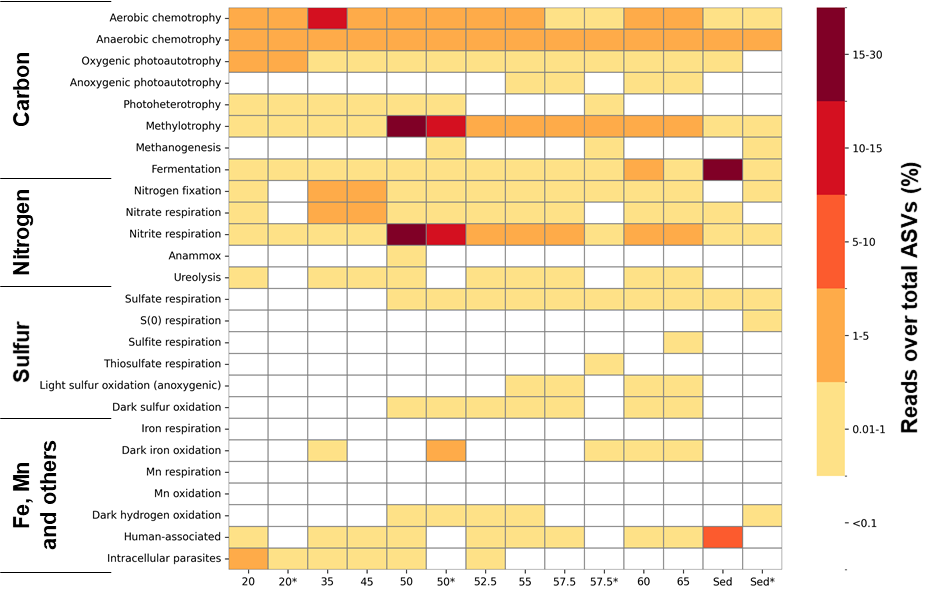


**Figure S6:** FAPROTAX functional prediction based on microbial community data of water column (depths indicated) and sediment (Sed) of Tasik Biru. Samples marked with an asterisk (*) indicate sequencing via universal primers, while the rest were sequenced with bacterial-specific primers.

**Table S1:** Detection limits for all analysis.

| **Technique** | **Dissolved species** | **Detection limit** |
| --- | --- | --- |
|  |  | **(µM)** |
| Ferrozine | Fe^2+^ and total Fe | 5 |
| Phosphomolybdate | PO_4_^3-^ | 0.1 |
| Molybdenum blue | Total silica | 1 |
| Cline assay | Sulfide | 1 |
| C/N analyzer | DOC | 80 |
|  | Total N | 16 |
| FIA | NH_4_^+^ | 1 |
|  | NO_3_^-^ | 1 |
|  | NO_2_^-^ | 1-2 |
| IC | SO_4_^2-^ | 60 |
|  | Cl^-^ | 300 |
|  | F^-^ | 3.2 |
|  | Br^-^ | 0.8 |
| ICP-MS | Na | 8.7 |
| (total dissolved) | Mg | 8.3 |
|  | Al | 8.3 |
|  | K | 1.5 |
|  | Ca | 5.1 |
|  |  | **(nM)** |
|  | V | 0.8 |
|  | Cr | 0.8 |
|  | Mn | 7.3 |
|  | Co | 3.4 |
|  | Ni | 3.3 |
|  | Cu | 3.3 |
|  | Zn | 6.1 |
|  | As | 0.5 |
|  | Se | 16.9 |
|  | Mo | 2.0 |
|  | Ag | 0.1 |
|  | Cd | 0.1 |
|  | Sb | 0.2 |
|  | Ba | 0.5 |
|  | Pb | 0.1 |
|  | U | 0.003 |

**Table S2:** Solid-phase contents of sediment (58 m water column depth) and soil (near the shore) from Tasik Biru.

| Sediment |  |  |  |  |  |  |  |  |  |  |  |  |
| --- | --- | --- | --- | --- | --- | --- | --- | --- | --- | --- | --- | --- |
| **Unit** | wt% | wt% | wt% | wt% | wt% | ppm | ppm | ppm | ppm | ppm | ppm | ppm |
| **Element** | **Fe** | **Al** | **Ca** | **K** | **Mg** | **As** | **Mn** | **Sb** | **Zn** | **V** | **Ba** | **Cr** |
| **Poorly crystalline  1 M HCl** | 2.91 | 0.27 | 1.01 | 0.01 | 0.03 | 81.82 | 1038.55 | 0.66 | 115.56 | 21.24 | 34.94 | 7.12 |
| **Crystalline  6 M HCl** | 1.34 | 0.16 | 0.03 | 0.01 | 0.01 | 0.00 | 105.54 | 262.55 | 126.38 | 12.61 | 3.45 | 6.93 |
| **Recalcitrant  Total extractable** | 1.49 | 3.94 | 0.15 | 0.43 | 0.26 | 947.08 | 105.58 | 9.50 | 89.04 | 60.59 | 57.94 | 33.74 |
| **Sum** | 5.74 | 4.37 | 1.19 | 0.45 | 0.29 | 1028.90 | 1249.67 | 272.70 | 330.99 | 94.44 | 96.33 | 47.79 |
| **Enrichment factor*** | 3.0 | 1.7 | 3.0 | 1.9 | 1.6 | 2.5 | 7.4 | 3.5 | 5.1 | 1.4 | 2.3 | 1.5 |
|  |  |  |  |  |  |  |  |  |  |  |  |  |
| **Unit** | ppm | ppm | ppm | ppm | ppm | ppm | ppm | ppm | ppm | ppm | ppm |  |
| **Element** | **Pb** | **Cu** | **Ni** | **Co** | **Th** | **Se** | **Mo** | **U** | **Tl** | **Ag** | **Cd** |  |
| **Poorly crystalline  1 M HCl** | 29.99 | 14.61 | 25.15 | 31.55 | 0.22 | 2.16 | 0.10 | 1.11 | 0.11 | 0.07 | 0.56 |  |
| **Crystalline  6 M HCl** | 16.40 | 39.24 | 17.31 | 13.28 | 0.88 | 0.00 | 1.49 | 0.02 | 0.15 | 2.60 | 0.68 |  |
| **Recalcitrant  Total extractable** | 25.82 | 33.15 | 30.72 | 17.59 | 2.28 | 6.33 | 0.51 | 0.55 | 0.30 | 0.38 | 0.09 |  |
| **Sum** | 72.21 | 87.00 | 73.18 | 62.41 | 3.38 | 8.49 | 2.10 | 1.67 | 0.56 | 3.05 | 1.32 |  |
| **Enrichment factor*** | 2.0 | 3.0 | 3.2 | 7.1 | 1.0 | 3.8 | 2.9 | 1.8 | 1.6 | 4.1 | 3.9 |  |
|  |  |  |  |  |  |  |  |  |  |  |  |  |
| Soil |  |  |  |  |  |  |  |  |  |  |  |  |
| **Unit** | wt% | wt% | wt% | wt% | wt% | ppm | ppm | ppm | ppm | ppm | ppm | ppm |
| **Element** | **Fe** | **Al** | **Ca** | **K** | **Mg** | **As** | **Mn** | **Sb** | **Zn** | **V** | **Ba** | **Cr** |
| **Poorly crystalline  1 M HCl** | 0.06 | 0.05 | 0.32 | 0.01 | 0.02 | 56.73 | 93.64 | 8.23 | 9.64 | 10.0 | 7.66 | 1.75 |
| **Crystalline  6 M HCl** | 0.12 | 0.04 | 0.00 | 0.00 | 0.00 | 71.66 | 13.23 | 10.72 | 1.50 | 4.7 | 0.14 | 2.12 |
| **Recalcitrant  Total extractable** | 1.70 | 2.51 | 0.08 | 0.23 | 0.17 | 282.09 | 61.91 | 60.00 | 54.19 | 54.8 | 33.54 | 28.17 |
| **Sum** | 1.88 | 2.60 | 0.40 | 0.24 | 0.19 | 410.47 | 168.79 | 78.95 | 65.34 | 69.59 | 41.33 | 32.04 |
|  |  |  |  |  |  |  |  |  |  |  |  |  |
| **Unit** | ppm | ppm | ppm | ppm | ppm | ppm | ppm | ppm | ppm | ppm | ppm |  |
| **Element** | **Pb** | **Cu** | **Ni** | **Co** | **Th** | **Se** | **Mo** | **U** | **Tl** | **Ag** | **Cd** |  |
| **Poorly crystalline  1 M HCl** | 11.68 | 9.02 | 5.08 | 2.17 | 0.24 | 0.49 | 0.08 | 0.49 | 0.04 | 0.20 | 0.18 |  |
| **Crystalline  6 M HCl** | 2.98 | 3.16 | 0.00 | 0.89 | 0.73 | 0.22 | 0.09 | 0.11 | 0.04 | 0.29 | 0.04 |  |
| **Recalcitrant  Total extractable** | 21.10 | 16.64 | 17.73 | 5.73 | 2.56 | 1.52 | 0.56 | 0.32 | 0.27 | 0.26 | 0.12 |  |
| **Sum** | 35.76 | 28.82 | 22.81 | 8.79 | 3.53 | 2.23 | 0.73 | 0.92 | 0.36 | 0.75 | 0.34 |  |

**Enrichment factor = sum in sediment/sum in soil*

**Table S3:** Saturation (SI) of minerals across water column depth as determined via Phreeqc modeling. Blue is undersaturated while red is oversaturated, with the color gradient indicative of the degree of saturation.

|  | **Mineral** | **Depth (m)** | | | | | | | | | | |
| --- | --- | --- | --- | --- | --- | --- | --- | --- | --- | --- | --- | --- |
|  |  | **10** | **20** | **27** | **35** | **45** | **50** | **52.5** | **55** | **57.5** | **60** | **65** |
| **Iron-bearing** | Ferrihydrite |  |  |  |  |  |  | 3.88 | 3.27 | 3.34 | 3.29 |  |
|  | K-Jarosite |  |  |  |  |  |  | 2.81 | 1.27 | 1.62 | 1.6 |  |
|  | Magnetite |  |  |  |  |  |  | 19.82 | 18.67 | 18.85 | 18.7 |  |
|  | Siderite |  |  |  |  |  |  | -0.72 | -0.53 | -0.49 | -0.50 | -0.46 |
|  | Strengite Fe(III)PO4 |  |  |  |  |  |  | 0.60 |  | 0.24 |  |  |
|  | Vivianite |  |  |  |  |  |  |  |  | -1.85 |  | -1.23 |
|  | Greenalite  (mixed Fe silicates) |  |  |  |  |  |  | -1.22 | -0.91 | -0.76 | -0.84 | -0.57 |
| **Sulfur-bearing** | Mackinawite |  |  |  |  |  |  |  | -0.04 | 0.00 | 0.18 | -0.14 |
|  | Pyrite |  |  |  |  |  |  |  | 23.77 | 23.77 | 24.13 | 23.53 |
|  | Sulfur |  |  |  |  |  |  |  | 11.05 | 11.01 | 11.19 | 10.91 |
|  | CoS(alpha) |  |  |  |  |  |  |  | 1.19 | 0.88 | 1.17 | 0.73 |
|  | CoS(beta) |  |  |  |  |  |  |  | 4.82 | 4.51 | 4.8 | 4.36 |
|  | NiS(alpha) |  |  |  |  |  |  |  | -1.29 | -1.67 | -1.46 | -1.82 |
|  | NiS(beta) |  |  |  |  |  |  |  | 4.21 | 3.83 | 4.04 | 3.68 |
|  | NiS(gamma) |  |  |  |  |  |  |  | 5.91 | 5.53 | 5.74 | 5.38 |
|  | Orpiment As_2_S_3_ |  |  |  |  |  |  |  |  | -1.91 | -1.11 |  |
|  | MoS_2_ |  |  |  |  |  |  |  | 0.72 | 0.68 | 1.36 | 0.04 |
| **Mn** | Rhodochrosite MnCO_3_ |  |  |  |  |  | -0.36 | 0.03 | -0.13 | -0.13 | -0.25 | -0.19 |
|  | Kutnahorite |  |  |  |  |  | 0.59 | 1.07 | 0.87 | 0.81 | 0.72 | 0.86 |
|  | MnHPO_4_ |  |  |  |  |  |  | 2.02 |  | 1.91 |  | 2.10 |
| **Others** | Quartz | -0.26 | -0.24 | -0.24 | -0.22 | -0.16 | 0 | 0.18 | 0.23 | 0.26 | 0.27 | 0.27 |
|  | Calcite | -0.46 | -1.09 | -0.43 | -0.52 | -0.54 | -0.76 | -0.66 | -0.71 | -0.77 | -0.75 | -0.66 |
|  | Hydroxylapatite | 4.41 | 1.47 | 4.55 | 4.26 | 4.28 |  | 0.19 |  | -0.42 |  | 0.91 |
|  | CoFe_2_O_4_ |  |  |  |  |  |  | 23.89 | 22.99 | 22.82 | 22.78 |  |
|  | SbO_2_ | -0.46 | 0.68 | -0.48 | -0.28 | -0.37 | 0.01 | -0.8 | -0.76 | -0.71 | -0.77 | -1.13 |
|  | Ba_3_(AsO_4_)_2_ | 9.89 | 8.45 | 9.96 | 9.81 | 9.92 | 9.51 | 10.18 | 10.87 | 11.07 | 10.9 | 11.06 |
|  | Barite | -0.82 | -0.79 | -0.79 | -0.79 | -0.8 | -0.89 | -0.79 | -0.5 | -0.39 | -0.38 | -0.37 |

**Table S4:** Comparison of geochemical parameters in the monimolimnion of Tasik Biru to other modern stratified lakes. The lakes are arranged from Fe-rich (left) to sulfide-rich (right).

| **Unit** | **Parameter** | **Brownie Lake, USA** | **Canyon Lake, USA** | **Lac Pavin, France** | **Lake Hall, USA** | **Lake Matano, Indonesia** | ***Tasik Biru*** | **Lake Poso, Indonesia** | **Fayetteville Green Lake, USA** |
| --- | --- | --- | --- | --- | --- | --- | --- | --- | --- |
| **m** | **Maximum depth** | 14 | 23 | 92 | 16 | 590 | *70* | 350 | 53 |
|  | **pH** | 6.9 |  | 6-6.4 | 6.5-6.7 | 7 | *6.9* | 7.5 | 7 |
| **uM** | **Fe(II)** | 1214-1605 | 1594 | <1200 | 13-750 | <150 | *23-54* | <35 | <40 |
| **uM** | **Mn** | 85 | 131 | <26 | 26-68 | 6-10 | *39-54* | 2-6 | 1-100 |
| **uM** | **Sulfide** | 19 | 2 | <27 | 1-100 | <0.1 | *<4* | - | <2000 |
| **uM** | **Sulfate** | 2.1 | <1 | 2-17 | 20-100 | <18 | *255* | 15-20 | 15000 |
| **uM** | **Si** | 368 | 6 | 300-1100 | - | - | *152-186* | 120-180 | - |
| **uM** | **Ca** | 2063 | 719 | 70-240 | 300-600 | - | *1350-1630* | 515-546 | 15000 |
| **uM** | **K** | 378 | 35 | 100-230 | 59 | - | *21-23* | 12-15 | - |
| **uM** | **Mg** | 877 | 120 | 70-330 | 200 | - | *125-157* | 75 | 2300 |
| **uM** | **Na** | 21646 | 256 | 250-460 | 292 | - | *82-83* | 60-75 | 1250-1700 |
| **uM** | **As** | - | - | 23-130 | 7-46 | - | *20-22* | 25 | - |
| **uM** | **PO_4_** | 20-46 | 1.2 | 1-336 | - | <1 | *<1.7* | <3 | - |
| **nM** | **Sb** | - | - | - | - | - | *6-12* | - | - |
| **nM** | **V** | - | - | 7-154 | 5-37 | - | *4-5* | 1-3 | - |
| **nM** | **Ba** | - | - | 5-841 | - | - | *313-378* | - | - |
| **nM** | **Ni** | - | - | 10-30 | 23-32 | 40 | *11-26* | 4-9 | 70-200 |
| **nM** | **Co** | - | - | 2-82 | 21-50 | 40 | *54-107* | 2-3 | 10-100 |
| **nM** | **Mo** | - | - | 5-10 | 0.7-1.3 | - | *2-4* | 0.5-0.7 | 13-17 |
| **nM** | **U** | 2.52 | - | 0.02-0.19 | - | - | *0.4-0.6* | 0.1 | - |
| **nM** | **Se** | - | - | - | - | - | *<62* | - | - |
| **nM** | **Cu** | <0.02 | <0.02 | 0.1-1.6 | 0.6-10 | - | *<3* | <1 | - |
| **nM** | **Zn** | <0.02 | 0.06 | 2-25 | 4-160 | - | *<18* | - | - |

References: (Balistrieri *et al.*, 1994; Michard *et al.*, 1994; Viollier *et al.*, 1995; Crowe *et al.*, 2008; Havig *et al.*, 2015; Lambrecht *et al.*, 2018; Cole *et al.*, 2020; Janssen *et al.*, 2024)

**References**

Balistrieri LS, Murray JW, Paul B (1994) The geochemical cycling of trace elements in a biogenic meromictic lake. *Geochimica et Cosmochimica Acta* 58, 3993–4008.

Breitwieser FP, Salzberg SL (2020) Pavian: Interactive analysis of metagenomics data for microbiome studies and pathogen identification. *Bioinformatics* 36, 1303–1304.

Cole DB, Planavsky NJ, Longley M, Böning P, Wilkes D, Wang X, Swanner ED, Wittkop C, Loydell DK, Busigny V, Knudsen AC, Sperling EA (2020) Uranium Isotope Fractionation in Non-sulfidic Anoxic Settings and the Global Uranium Isotope Mass Balance. *Global Biogeochemical Cycles* 34.

Crowe SA, O’Neill AH, Katsev S, Hehanussa P, Douglas Haffner G, Sundby B, Mucci A, Fowle DA (2008) The biogeochemistry of tropical lakes: A case study from Lake Matano, Indonesia. *Limnology and Oceanography* 53, 319–331.

Havig JR, McCormick ML, Hamilton TL, Kump LR (2015) The behavior of biologically important trace elements across the oxic/euxinic transition of meromictic Fayetteville Green Lake, New York, USA. *Geochimica et Cosmochimica Acta* 165, 389–406.

Janssen DJ, Damanik A, Tournier N, Tolu J, Winkel L, Cahyarini SY, Vogel H (2024) Biogeochemical cycling of trace elements and nutrients in ferruginous waters: Constraints from a deep oligotrophic ancient lake. *Limnology and Oceanography* 69, 2775–2790.

Lambrecht N, Wittkop C, Katsev S, Fakhraee M, Swanner ED (2018) Geochemical Characterization of Two Ferruginous Meromictic Lakes in the Upper Midwest, USA. *Journal of Geophysical Research: Biogeosciences* 123, 3403–3422.

Michard G, Viollier E, Jézéquel D, Sarazin G (1994) Ceochemical study of a crater lake: Pavin Lake, France - Identification, location and quantification of the chemical reactions in the lake. *Chemical Geology* 115, 103–115.

Viollier E, Jézéquel D, Michard G, Pépe M, Sarazin G, Alberic P (1995) Geochemical study of a crater lake (Pavin Lake, France): Trace-element behaviour in the monimolimnion. *Chemical Geology* 125, 61–72.
